# Understanding Pediatric Surgery Cancellation: Geospatial Analysis

**DOI:** 10.2196/26231

**Published:** 2021-09-10

**Authors:** Lei Liu, Yizhao Ni, Andrew F Beck, Cole Brokamp, Ryan C Ramphul, Linda D Highfield, Megha Karkera Kanjia, J “Nick” Pratap

**Affiliations:** 1 Division of Biomedical Informatics Cincinnati Children’s Hospital Medical Center Cincinnati, OH United States; 2 Department of Biomedical Informatics University of Cincinnati Cincinnati, OH United States; 3 Department of Pediatrics University of Cincinnati Cincinnati, OH United States; 4 Division of General and Community Pediatrics Cincinnati Children’s Hospital Medical Center Cincinnati, OH United States; 5 Division of Hospital Medicine Cincinnati Children’s Hospital Medical Center Cincinnati, OH United States; 6 Division of Biostatistics and Epidemiology Cincinnati Children’s Hospital Medical Center Cincinnati, OH United States; 7 Department of Government Relations and Community Benefits Texas Children’s Hospital Houston, TX United States; 8 Department of Management Policy & Community Health University of Texas Health Science Center School of Public Health Houston, TX United States; 9 Department of Epidemiology Human Genetics & Environmental Sciences University of Texas Health Science Center School of Public Health Houston, TX United States; 10 Department of Pediatric Anesthesiology and Pain Management Texas Children’s Hospital Houston, TX United States; 11 Department of Anesthesiology Baylor College of Medicine Houston, TX United States; 12 Department of Anesthesia Cincinnati Children's Hospital Medical Center Cincinnati, OH United States

**Keywords:** surgery cancellation, socioeconomic factors, spatial regression models, machine learning

## Abstract

**Background:**

Day-of-surgery cancellation (DoSC) represents a substantial wastage of hospital resources and can cause significant inconvenience to patients and families. Cancellation is reported to impact between 2% and 20% of the 50 million procedures performed annually in American hospitals. Up to 85% of cancellations may be amenable to the modification of patients’ and families’ behaviors. However, the factors underlying DoSC and the barriers experienced by families are not well understood.

**Objective:**

This study aims to conduct a geospatial analysis of patient-specific variables from electronic health records (EHRs) of Cincinnati Children’s Hospital Medical Center (CCHMC) and of Texas Children’s Hospital (TCH), as well as linked socioeconomic factors measured at the census tract level, to understand potential underlying contributors to disparities in DoSC rates across neighborhoods.

**Methods:**

The study population included pediatric patients who underwent scheduled surgeries at CCHMC and TCH. A 5-year data set was extracted from the CCHMC EHR, and addresses were geocoded. An equivalent set of data >5.7 years was extracted from the TCH EHR. Case-based data related to patients’ health care use were aggregated at the census tract level. Community-level variables were extracted from the American Community Survey as surrogates for patients’ socioeconomic and minority status as well as markers of the surrounding context. Leveraging the selected variables, we built spatial models to understand the variation in DoSC rates across census tracts. The findings were compared to those of the nonspatial regression and deep learning models. Model performance was evaluated from the root mean squared error (RMSE) using nested 10-fold cross-validation. Feature importance was evaluated by computing the increment of the RMSE when a single variable was shuffled within the data set.

**Results:**

Data collection yielded sets of 463 census tracts at CCHMC (DoSC rates 1.2%-12.5%) and 1024 census tracts at TCH (DoSC rates 3%-12.2%). For CCHMC, an L2-normalized generalized linear regression model achieved the best performance in predicting all-cause DoSC rate (RMSE 1.299%, 95% CI 1.21%-1.387%); however, its improvement over others was marginal. For TCH, an L2-normalized generalized linear regression model also performed best (RMSE 1.305%, 95% CI 1.257%-1.352%). All-cause DoSC rate at CCHMC was predicted most strongly by *previous no show*. As for community-level data, the proportion of African American inhabitants per census tract was consistently an important predictor. In the Texas area, the proportion of overcrowded households was salient to DoSC rate.

**Conclusions:**

Our findings suggest that geospatial analysis offers potential for use in targeting interventions for census tracts at a higher risk of cancellation. Our study also demonstrates the importance of home location, socioeconomic disadvantage, and racial minority status on the DoSC of children’s surgery. The success of future efforts to reduce cancellation may benefit from taking social, economic, and cultural issues into account.

## Introduction

### Background

Surgical interventions, along with other diagnostic and therapeutic procedures performed under anesthesia, can deliver significant health benefits; it has been estimated that 30% of the global burden of disease is treatable by surgery [1]. Unlike most drug and nonprocedural therapies that require ongoing adherence for maximal benefit, surgery is typically delivered at a single encounter with a complex multidisciplinary health care team. Therefore, barriers to compliance with surgery are likely to be different from barriers to chronic treatment compliance.

Cancellation is an important barrier to the successful delivery of surgical therapy and is reported to affect between 2% and 20% of the 50 million procedures performed annually in American hospitals [2,3]. Cancellation has become a focus of interest at children’s hospitals in view of its substantial negative repercussions for patients, families, and institutions. First, if surgery is canceled, the child fails to receive therapeutic or diagnostic benefits. Taking as an example the most common surgery in childhood, insertion of *ear tubes,* confirmed by a systematic review, reduces hearing loss in children with otitis media with effusion [4]. Cancellation of, or even delay in, insertion of ear tubes may thus impair language or speech development and affect behavioral, cognitive, or quality of life outcomes. For families, surgery cancellation leads to psychological stress and increased financial burden. As an illustration, researchers at another academic tertiary children’s hospital, also in the American Midwest, found an average wasted round-trip of more than 160 miles for those who come for surgery but had to cancel [5]. This resulted in one-third of accompanying family members missing a day of work, which was unpaid in half of the cases. Parents and children expressed disappointment, frustration, and anger as a result of cancellation. From an institutional perspective, expensive staff and facilities costs are not reimbursed when surgeries are canceled. Even with a low 4.1% day-of-surgery cancellation (DoSC) rate at our hospital, potential lost revenue exceeds US $3000 per hour for operating room billing alone, with more than 5 hours lost per day, costing over US $2 million per year [6].

In our previous work, we found that up to 85% of cancellations may be amenable to modification of patients’ and families’ behaviors. We undertook a quality improvement project that reduced cancellations by delivering interventions *across the board* to all scheduled patients [6]. In preparation for subsequent improvement efforts, we sought predictors of cancellation to gain insight into its etiology and with the aim of targeting future efforts more efficiently [7].

To date, most studies on surgery cancellation applied classical statistical techniques to demonstrate association [8-14], whereas few studies have used machine learning to predict surgical cases at risk of cancellation [7,15,16]. Most recently, we developed machine learning–based approaches to identify *individual surgery cases* at high risk of DoSC from patient-specific and contextual data from 2 distinct pediatric surgical sites of Cincinnati Children’s Hospital Medical Center (CCHMC), offering the promise of targeted interventions [7]. At the conception of this study, we hypothesized that the risk of DoSC at our tertiary children’s hospital varies according to the location of the patient. We sought *geographical clusters* of high and low cancellation rates and used these findings to explore the underlying social determinants.

In recent years, increasing volumes of geospatial data have become publicly available, including from censuses, cataloging crimes, and relating to a variety of social and economic processes. As, in spatial data sets, observations may not be independent (spatial autocorrelation) or the relationships between variables may vary across geographical space (spatial nonstationarity), assumptions underlying conventional statistical modeling approaches may be violated. For these reasons, specialized methodologies have been developed for geospatial analysis to explain the spatial patterns of human behavior and the underlying factors that contribute to or explain these patterns [17]. In addition, geospatial models may capture spatial patterns (eg, spatial clusters) during model construction to achieve a better fit [18-20]. Spatial autocorrelation measures quantify the correlation of a variable with itself through geographical space [21]. A mixed regressive spatial autoregressive (SAR) model combines an autoregressive structure with a conventional regression model by assuming that a regional outcome is also impacted by outcomes from its neighbors [18], whereas a spatial error model (SEM) combines a conventional regression model with spatially autoregressive disturbances [22,23]. Spatial moving average models are similar to SEMs but use a different autocorrelation structure to represent the disturbances. Spatial Durbin models extend the SAR framework by hypothesizing that a regional outcome is additionally impacted by predictors from the neighborhoods that comprise it [18,23]. Similarly, an SAR confused model is a generalized SAR model that accommodates spatial dependence in both the outcome variable and error terms [18]. A spatial Durbin error model includes exogenous interaction effects in addition to interaction effects among error terms [23].

Geospatial modeling approaches have been applied to a wide range of public health problems, such as the estimation of mortality or of air pollution effects or identification of causal factors in disease [24-26]. However, such spatial analytical techniques have not been used to study the geography of surgery cancellation. In particular, community characteristics provide vital information that is lacking when using individual-level data in isolation. Therefore, in this study, we explore a variety of established geospatial models to identify and visualize spatial trends in cancellation rates and candidate predictors. The findings were compared with those of the generalized linear regression model (GLM) and deep learning model.

### Objectives

This study presents a geospatial analysis of patient-specific variables from the electronic health records (EHRs) of CCHMC and of Texas Children’s Hospital (TCH) as well as of socioeconomic factors measured at the census tract level. We use the data to understand the potential contributors to spatial variation in the cancellation rates of pediatric surgery. We hypothesize that there are marked disparities in DoSC rates across neighborhoods. To our knowledge, this is the first study to investigate the geographic variation of pediatric surgery cancellation rates. The long-term objective is to understand contributors underlying DoSC and barriers experienced by patients and their families so that support can be focused efficiently toward families who are both in need and are most likely to benefit.

## Methods

### Data

A 5-year geocoded data set (May 2011-May 2016) of 88,013 surgeries including 3702 (4.21%) DoSCs corresponding to patients living in the hospital’s primary service area was extracted from the CCHMC EHR. The data set included 2 primary surgical sites (main and Liberty campuses) of the institution that covers 472 census tracts in Greater Cincinnati. An equivalent set of 166,533 surgeries over 5.7 years with 10,236 (6.14%) DoSCs was extracted from the TCH EHR for validation. The data set included 3 primary surgery sites (Texas Medical Center, West Campus, and the Woodlands) of the institution that serves 1065 census tracts in Greater Houston. Ethics approval for this study was provided by the CCHMC institutional review board (study ID 2018-4568). Both CCHMC and TCH are urban, pediatric academic medical centers that function as the primary pediatric facilities for their surrounding metropolitan areas and also accept many tertiary and quaternary care referrals from elsewhere. All home locations were geocoded with an in-house geographic information system to ensure that no protected health information was sent outside the institution. Owing to high address matching accuracy, 90.2% (229,600/254,546) of the locations were geocoded at the city-block level (ie, a group of buildings surrounded by streets), and a further 6.8% (17,309/254,546) were geocoded at street level (ie, center of the matched street). For all surgical activities at CCHMC, cancellations are comprehensively adjudicated to one of 10 reason codes by clinical staff at the time of cancellation, thus allowing analysis for specific causes, including acute patient illness, failure to attend surgery (*no show*), failure to comply with eating and drinking instructions (*nil per os* [*NPO*] *violation*), and refusal to undergo surgery by either patient or family. For CCHMC, rescheduled cases were defined as completed surgeries with prior cancellations of similar case length within the preceding 90 days for the same individual. At TCH, in the absence of estimated case length data, rescheduled cases were determined by the procedure name and service department. All rescheduled cases (CCHMC: 1578/88,013, 1.79%; TCH: 4077/166,533, 2.45%) were excluded from the analysis to avoid diluting the effects of cancellation predictors by subsequently completed surgeries.

EHR variables for individual surgery cases, including recent health care use, schedule-related factors, prior cancellation behaviors, and information from a preoperative telephone call, were extracted as previously described [7] and spatially aggregated at the census tract level (Table 1). The estimated driving time from patients’ homes to surgical sites was calculated and categorized into 6-minute intervals (*>60* for locations farther than 1 hour away) using the DeGAUSS R package (Cole Brokamp) [27]. Socioeconomic factors were obtained from the US Census Bureau’s 2011-2015 American Community Survey (ACS) 5-year estimates, which provide data at the level of individual census tracts [28]. ACS variables were selected for practical relevance to successful preparation and attendance for surgery, including those relating to poverty, home ownership, household vehicle availability, housing (vacancy, value, and crowding), marriage, educational attainment, population density, linguistic isolation, African American (*Black*) race, and Hispanic heritage (Table 2). Census tract population density was computed as the ratio of the population to the total land area.

**Table 1:** Case-based variables from institutional electronic health records. (N=14).

Category	Variables, n (%)	Description
Transportation	1 (7)	Driving time from home to the surgical site
Preoperative phone call	1 (7)	Number of call attempts
Recent health care use	5 (35)	Number of recent emergency room attendance (two 2 time points), number of medications taken regularly at home before surgery, office visits, and hospitalizations in the previous 6 months
Prior cancellation behaviors	5 (35)	Numbers of previous cancellations, previous *no shows*, previous other cancellations, clinic *no shows*, and previous surgeries
Surgery related factors	2 (14)	Lead time and estimated case length

**Table 2:** American Community Survey data from the US Census Bureau measured at the census tract level.

ACS^a^ table code	Table description	Extracted data
B02001	Race	Black or African American race
B03003	Hispanic or Latino origin	Hispanic or Latino heritage
B17012	Poverty status of families by household type by number of related children aged <18 years	Families in poverty
B15002	Sex by educational attainment for the population aged ≥25 years	Population with low educational attainment
B16002	Language spoken at home and ability to speak English	Linguistic isolation
B06008	Place of birth by marital status in the United States	Adults never married
B08201	Household size by vehicles available	No car in household
B25003	Residential tenure	Rented houses
B25077	Median home value (US $)	Median home value
B19125	Median family income in the past 12 months by the presence of own children aged <18 years	Median household income
B25002	Residential occupancy status	Vacant houses
B25014	Tenure by occupants per room	Household overcrowding
B01003	Census tract total population	Total population

^a^ACS: American Community Survey.

### Spatial Autocorrelation

In geospatial analysis, it is important to assess the spatial independence of variables before model construction. Spatial autocorrelation measures describe the degree of spatial dependence or patterns for a variable across a spatial area [21]. We used the global Moran I statistic to test spatial independence for the DoSC rate and extracted variables [29]. Moran I values with significant *P* values (*P*<.05) indicate that the values for a variable are either spatially clustered (positive Moran I value) or dispersed (negative Moran I value), whereas there is no spatial dependence of the variable if the *P* value is not significant.

### Data Processing

DoSCs resulting from CCHMC’s top four most frequent patient-related cancellation reasons (ie, patient illness, *no show*, NPO violation, and patient or family refusal) were considered as canceled cases (denoted as all-cause cancellation) [7]. Census tracts without inhabitants (eg, for CCHMC, the census tract corresponding to the Cincinnati or Northern Kentucky International Airport—GEOID 21015980100) or with less than 20 surgical cases were excluded a priori. The rate of DoSC was calculated per census tract for the primary service area of the 2 hospitals (463 for CCHMC and 1024 for TCH) with empirical Bayesian shrinkage toward a beta before lessening the influence of sparsely populated tracts with few patients [30]. The corresponding rates for common patient-related cancellation reasons were also computed for CCHMC (but not for TCH for which such categorization was not available). For each census tract, the rates of the categorized driving times were similarly computed using empirical Bayesian estimation. The most common category of driving time was used as the base category to avoid the linear dependencies induced between the features. Missing ACS values (ie, median home value: 0.9% (4/463) of missing values for CCHMC and 1.66% (17/1024) of missing values for TCH; median household income: 6.7% (31/463) of missing values for CCHMC and 2.8% (29/1024) of missing values for TCH) were imputed using grand mean and mode imputation. The median home value and the median household income were categorized based on information from the US Census Bureau website [31-34]. All variables based on percentages were rescaled based on IQR to aid the interpretation of regression models [35]. The collinearity among variables was tested using the variance inflation factor (VIF) [36]. Variables not exceeding a predefined threshold of collinearity (VIF<10) were included in the model construction. Finally, EHR and ACS variables without evidence of significant geospatial clustering were excluded from the data set.

### Spatial Weights

Spatial weight matrices summarize the spatial relations between the census tracts. Neighboring tracts were determined by sharing at least one boundary edge. Inverse distance weighting was applied to compute spatial weights [37], and weight matrices were then standardized by row, such that the sum of spatial weights for each census tract equals 1.

### Conventional Regression Models

We modeled the prediction of the DoSC rate for each census tract as a supervised regression problem and tested both nonspatial regression models (GLM, L2-normalized GLM, support vector machine with polynomial kernels [SVM-P], and decision tree) and spatial regression models including SAR model, spatial Durbin model, SEM, spatial Durbin error model, spatial moving average, and SAR confused models [18,22,23,38-40]. Appropriate variants of the spatial regression models, such as the L2-normalized SAR models, were also implemented. We used these models to allow for the possibility of spatial impact on a census tract by neighboring tracts. Regression models were implemented using packages for the R programming language (R Foundation for Statistical Computing) [41].

### Deep Learning Models

**Figure 1:**
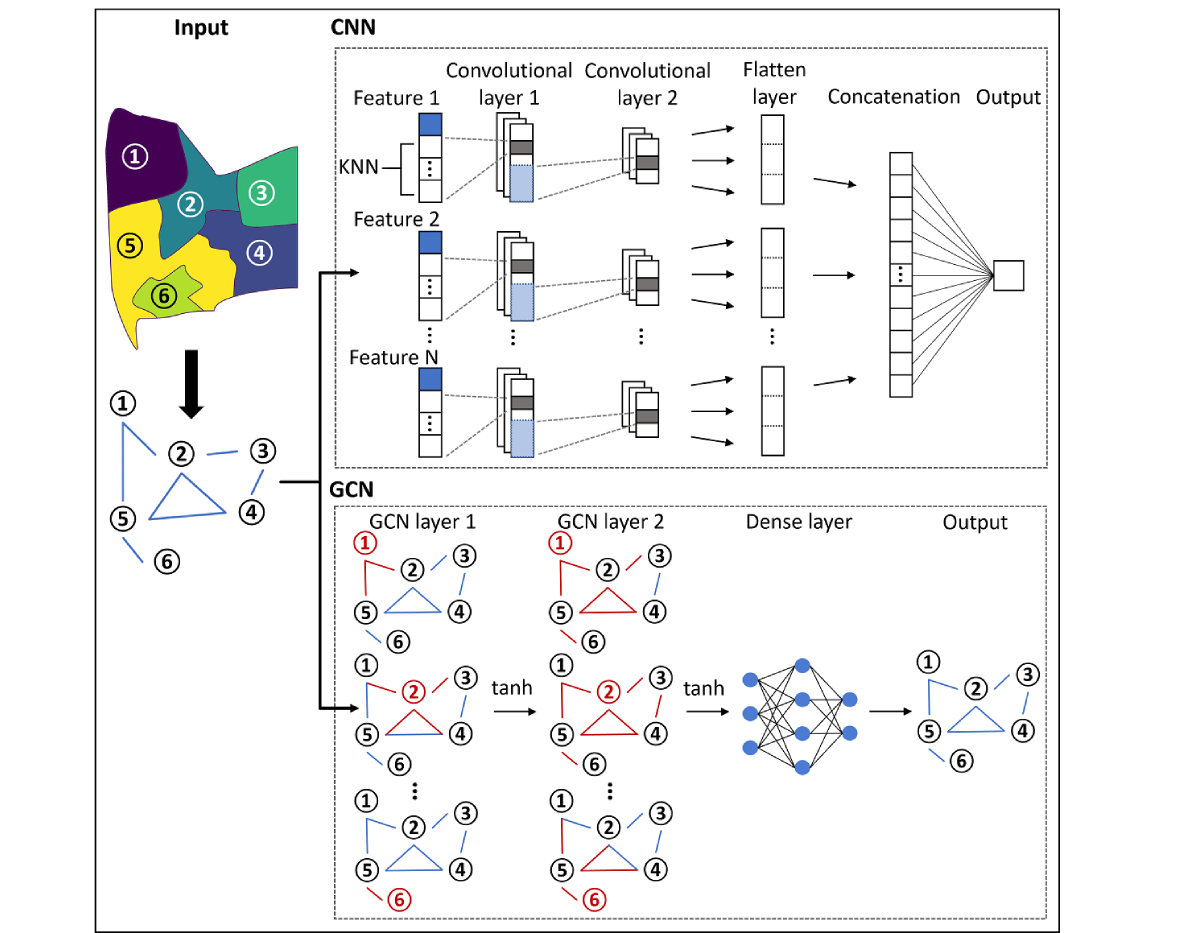
Model development for deep learning models. CNN: convolutional neural networks; GCN: graph convolutional networks; KNN: K-nearest neighbor.

In addition to traditional regression models, we implemented convolutional neural networks (CNNs) and graph convolutional networks (GCNs) to allow for the possibility of nonlinear relationships between the variables and DoSC rates [42,43]. Figure 1 illustrates the structures of the CNN and GCN models. For CNNs, targeted census tracts with their K-nearest neighbors (K=5, 10, 15, and 20) were used to construct feature vectors, each of which was trained by a 2-layer 1D CNN. The concatenated output was then used to predict the DoSC rates for individual census tracts. For GCNs, an adjacency matrix (eg, inverse distance weighting matrix) representing the graph structure and a feature matrix were taken as inputs and a framework with two layers of GCN and 1 layer of fully connected neural network to predict DoSC rates. Deep learning models were implemented using TensorFlow (version 2.2) for Python [44,45].

### Experimental Setup

Owing to the relatively low number of census tracts (sample size) in the data sets, a nested 10-fold cross-validation (10 folds for both the outer and inner loops) was adopted. The approach randomly split the data set into 10 rotating subsets: 9 for model training and hyperparameter tuning and 1 for testing at each run. At each iteration, an inner cross-validation was applied to the 9 folds of training data to tune a model’s hyperparameters with grid search parameterization [46], including (1) cost parameters for L2-normalized GLM [38], L2-normalized SAR model [18], and SVM-P [39] (screened from 10^−6^ to 10^6^); (2) optimal degree for SVM-P (screened from 1 to 3); (3) minimum number of observations in a node (3, 5, 10, 15, and 20) and the complexity parameters (screened from 10^−6^ to 10^−1^, 0.3, 0.5, and 0.8) for decision tree [40]; (4) learning rates of an optimizer parameter (screened from 10^−3^ to 10^−1^) for deep learning models [42,43]; (5) filter size of kernels (2 and 3) for 1D CNN layers [42]; and (6) dimensionality of output space (40, 50, 60, 75, and 90) for 2 GCN layers [43]. The model with optimal hyperparameters was then trained on all 9 folds of data and evaluated on the hold-out subset. The process was repeated 10 times to cover all subsets, and the evaluation score was averaged across the subsets as the final performance of the model. Model selection was based on the performance in the outer loop of the nested cross-validation. For models without hyperparameters, a 10-fold cross-validation was performed using the same folds as used in the outer loop of nested cross-validation. To assess the validity of the geospatial analysis, we compared the model performances with those aggregated from individual DoSC predictions. The best-performing gradient boosted logistic regression model, with 58 EHR variables developed in our previous study, was applied to the surgical cases located within the studied census tracts to predict DoSC [7]. The individual-level predictions were then aggregated to predict the DoSC rate per census tract (denoted as the individual-prediction-aggregation model).

### Evaluation Metrics

Model performance was evaluated by root mean square error (RMSE), which is a commonly used evaluation metric for numerical predictions in regression analysis [47]. The spatial autocorrelation of model residuals was assessed using the global Moran I statistic for indications that the model was misspecified. Geographically, weighted Pearson correlation was used to mark census tracts with significant local correlations between observed and predicted cancellation rates [48]. A permutation-based technique was used to calculate the feature importance scores using the DALEX (Model Agnostic Language for Exploration and Explanation) R package (Przemyslaw Biecek) [49]. The importance of every variable was measured by computing the increment of the RMSE when a single variable was shuffled within the data set.

## Results

### Descriptive Statistics for the Data Sets

Among 86,435 CCHMC surgical cases meeting the analysis selection criteria, the overall all-cause DoSC rate was 3.76% (3255). Patients lived in 472 different census tracts within the primary service area of the CCHMC. Of the 472 census tracts, 9 (1.9%) contributed to a few surgical cases (<20 cases) and were excluded from the analysis. Of the 463 remaining, the 97.5th percentile for cancellation rate was 9.4%, but 10 tracts (2.2%) had no cancellations. The TCH data set contained 166,533 surgical cases over 5.7 years, corresponding to 1065 different census tracts, with a DoSC rate of 6.14% (10,236/166,533). After similar preprocessing, 162,026 surgery cases and 1024 census tracts, with a median DoSC rate of 6.2%, were included in the analysis.

Of note, the 463 census tracts within the CCHMC study area had a median population of 3987 (IQR 2668, maximum 20,188), with a total population of 2 million. The 1024 Houston area census tracts encompassed a population of 6.38 million (median 5342 per tract). The relatively small population per census tract supports that, although the ACS provides aggregate statistics, these represent features of the locale and community immediately adjacent to patients’ homes with a high degree of spatial granularity. In support of this assertion, the proportion of African American patients in each census tract from the CCHMC EHR data is closely associated with the equivalent proportion of the general population in the ACS data (*R*^2^=0.89). This finding underscores the validity of using ACS variables as surrogates for individual patients’ socioeconomic and minority status, in addition to their characterization of the surrounding context.

**Figure 2:**
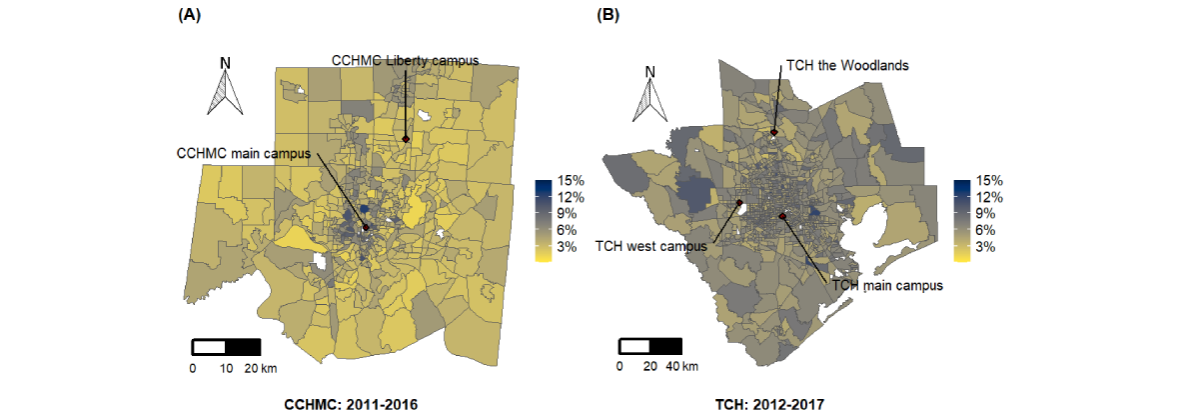
Geospatial distributions of day-of-surgery cancellation rate in the primary service areas of (A) Cincinnati Children’s Hospital Medical Center (2011-2016) and (B) Texas Children’s Hospital (2012-2017). CCHMC: Cincinnati Children’s Hospital Medical Center; TCH: Texas Children’s Hospital.

**Figure 3:**
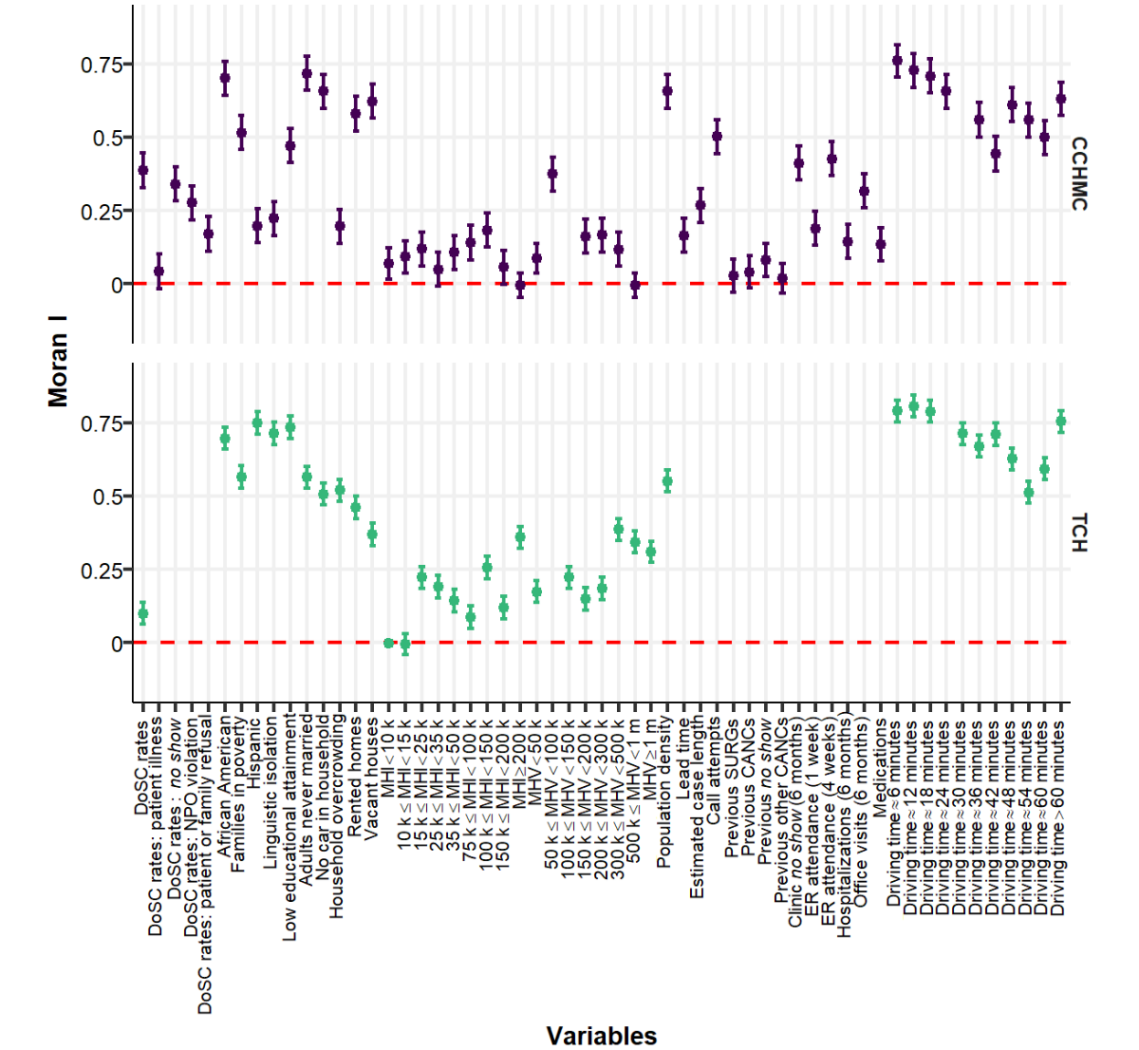
Spatial autocorrelation of day-of-surgery cancellation rate, case-based electronic health record variables and American Community Survey variables for Cincinnati Children’s Hospital Medical Center and Texas Children’s Hospital measured by global Moran I with 95% CI. CANC: cancellation; CCHMC: Cincinnati Children’s Hospital Medical Center; DoSC: day-of-surgery cancellation; ER: emergency room; MHI: median household income; MHV: median house value; NPO violation: failure to comply with eating and drinking instructions; SURG: surgery; TCH: Texas Children’s Hospital.

Figure 2 depicts the geospatial variation in DoSC rate by census tract of home location in the Greater Cincinnati and Houston regions. Enlarged maps are presented in Figures S1 and S2 in Multimedia Appendix 1, and interactive maps are presented in Multimedia Appendix 2 [50]. For CCHMC, tracts with increased cancellation risk clustered mainly in the most populous urban areas. Tracts with lower cancellation risk were located in suburban and rural locations. However, in the Houston area (Figure 2), high- and low-canceling census tracts were more geographically dispersed. These visual impressions are supported by the global Moran I as a measure of spatial autocorrelation (Figure 3).

### Predicting DoSC Rates With Conventional Regression and Deep Learning Models

**Figure 4:**
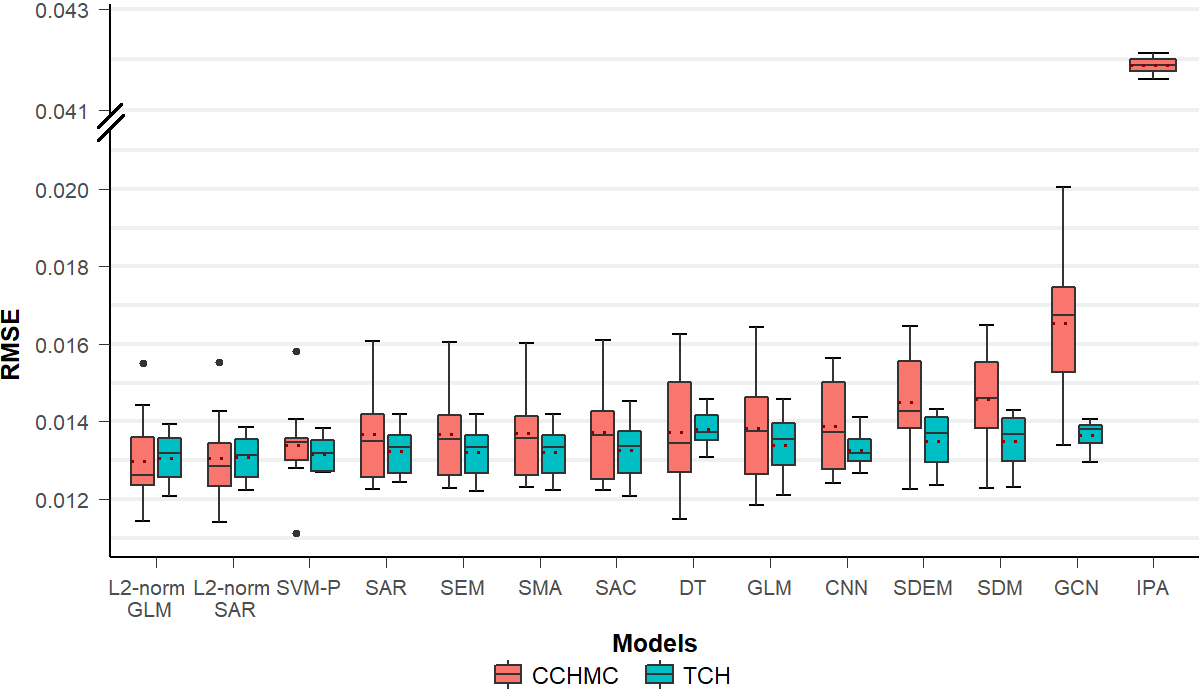
Model performance for predicting day-of-surgery cancellation rates at census tract level for Cincinnati Children’s Hospital Medical Center and Texas Children’s Hospital. Dashed line in each box represents the mean value of root mean squared error. CCHMC: Cincinnati Children’s Hospital Medical Center; CNN: convolutional neural networks; DT: decision tree; GCN: graph convolutional networks; GLM: generalized linear regression model; IPA: individual-prediction-aggregation model; RMSE: root mean square error; SAC: spatial autoregressive confused model; SAR: spatial autoregressive model; SDEM: spatial Durbin error model; SDM: Spatial Durbin models; SEM: spatial error model; SMA: spatial moving average; SVM-P: support vector machine with polynomial kernels; TCH: Texas Children’s Hospital.

In preparation for model development, we tested for collinearity among case-based EHR variables and ACS independent variables using VIF. None of the variables exceeded a predefined threshold of collinearity (VIF<10), which supports their independence. Certain variables were excluded from model development because they had insignificant Moran I values in the spatial autocorrelation analysis (Figure 3). Specifically, these variables excluded for low spatial variation were numbers of previous surgeries (*P*=.13), previous cancellations (*P*=.06), previous non–*no show* cancellations (*P*=.19), and patient illness (*P*=.07). The exclusion resulted in 46 and 37 numerical variables for model construction for CCHMC and TCH, respectively. Figure 4 presents the performance of the regression and deep learning models in predicting the all-cause DoSC rates. The lowest (best) RMSE was generated by the L2-normalized GLM at 1.299% (95% CI 1.21%-1.387%) for the CCHMC data set. All models outperformed the individual-prediction-aggregation model (RMSE 4.189%, 95% CI 4.178%-4.201%). This finding was statistically significant (*P*<.001). For the TCH data set, L2-normalized GLM also achieved the best performance, yielding an RMSE of 1.305% (95% CI 1.257%-1.352%).

**Figure 5:**
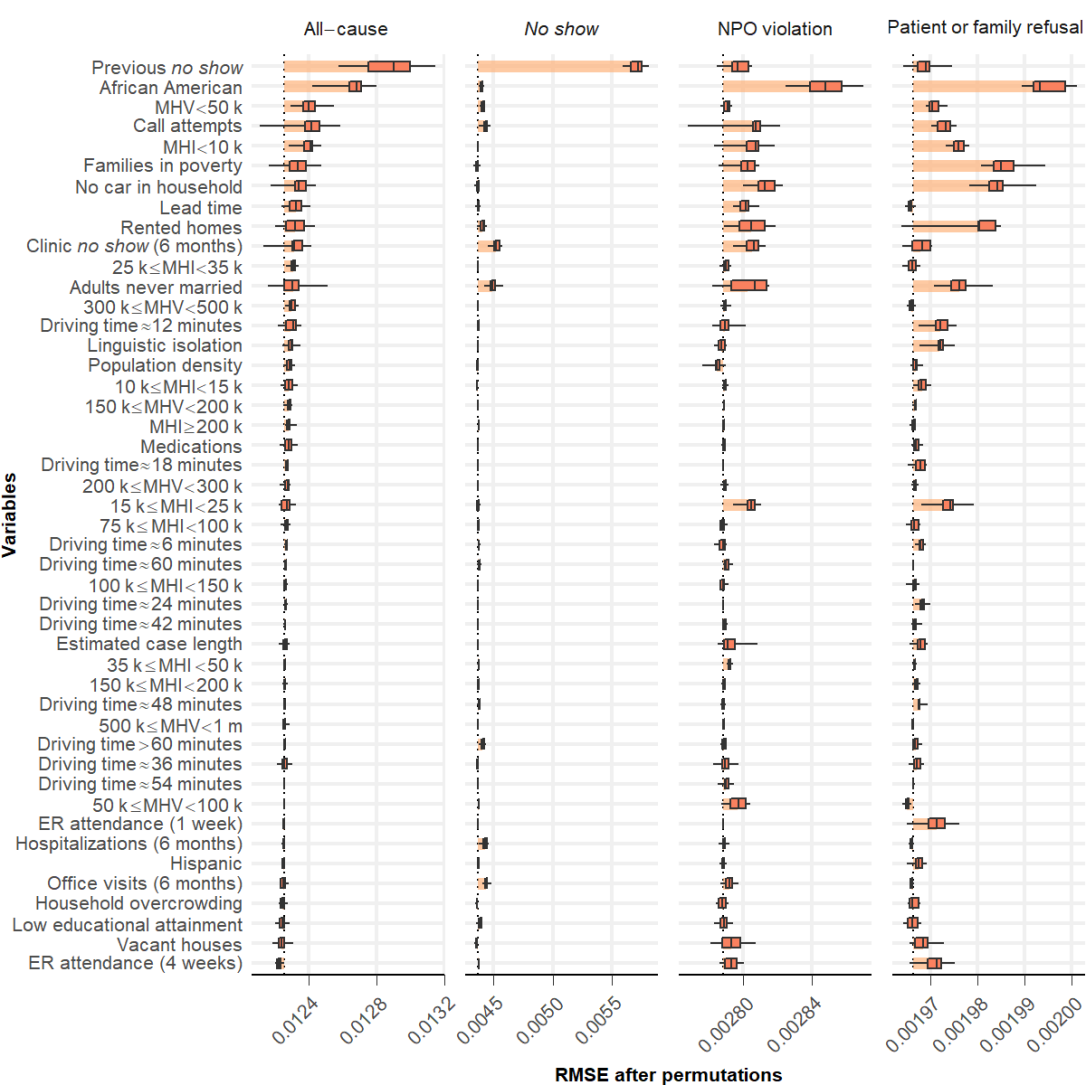
Feature importance generated from the best-performing L2-normalized generalized linear regression model for the Cincinnati Children’s Hospital Medical Center data set. Variables were ranked in descending order based on their importance in predicting all-cause day-of-surgery cancellation rates. ER: emergency room; MHI: median household income; MHV: median house value; NPO: nil per os; RMSE: root mean squared error.

**Figure 6:**
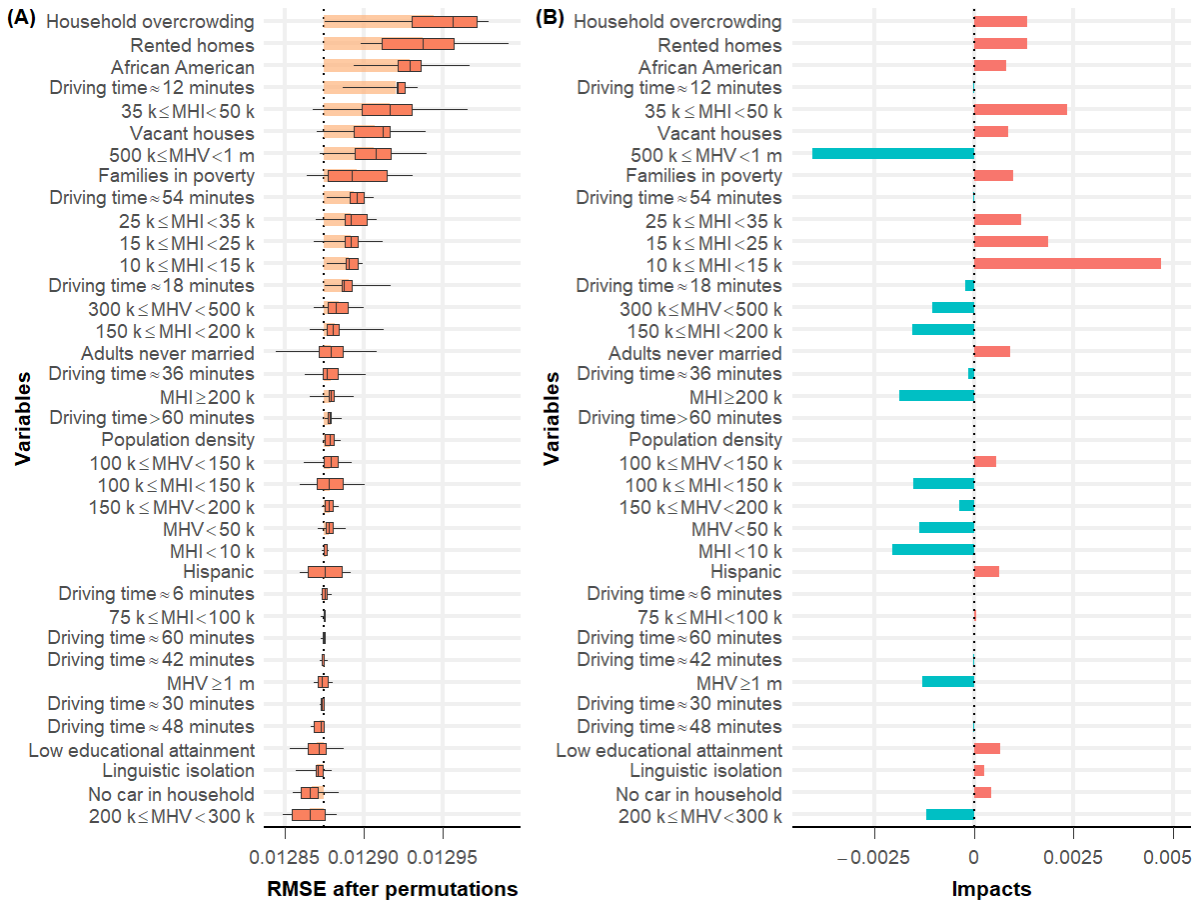
(A) Feature importance and (B) impacts generated from the best-performing L2-normalized generalized linear regression model for the Texas Children’s Hospital data set. Variables were ranked in descending order based on their importance in predicting day-of-surgery cancellation rates. MHI: median household income; MHV: median house value; RMSE: root mean squared error.

To augment our understanding of potential cancellation causes, we used DALEX [49] to identify the most important predictors in the best-performing L2-normalized GLMs (Figures 5 and 6). All-cause cancellation at CCHMC was predicted most strongly by the variable *previous no show* (Figure 5). Turning to community-level variables, the proportion of African American inhabitants per census tract is important. However, among community-level factors in the Houston area, the proportion of overcrowded households showed the strongest association with surgery cancellation rate (Figure 6). The median household income was also predictive, whereas spatial models highlighted the importance of clustered neighborhoods with low educational attainment (Figure S3 in Multimedia Appendix 1).

**Figure 7:**
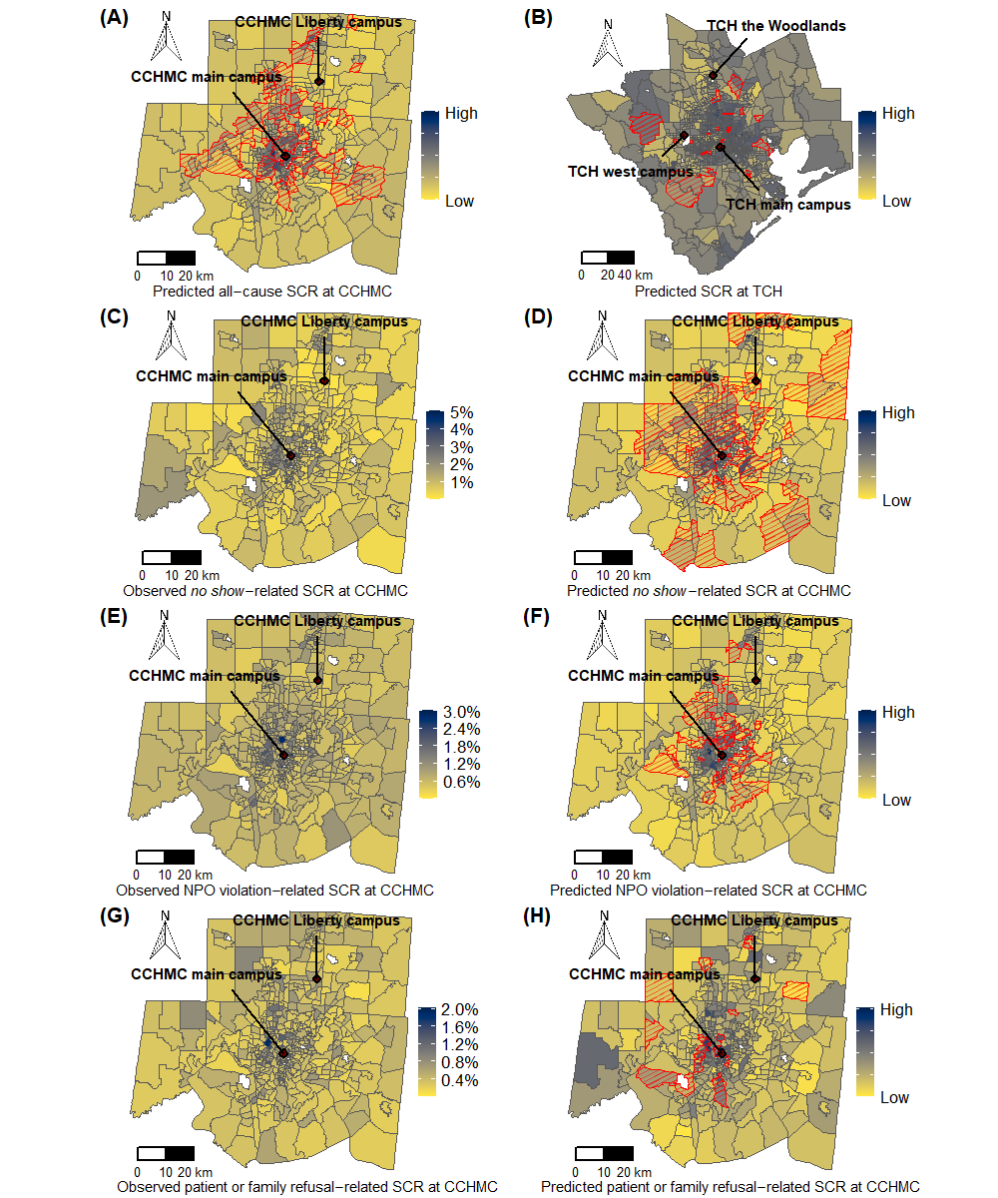
Comparison of surgery cancellation rates at census tract level predicted by the best-performing model to actual data. Census tracts with significant local correlation between the observed and predicted values are marked with red borders and cross-hatching. (A) Predicted all-cause surgery cancellation rate at Cincinnati Children’s Hospital Medical Center; (B) predicted surgery cancellation rate at Texas Children’s Hospital; (C) observed no show–related surgery cancellation rate at Cincinnati Children’s Hospital Medical Center; (D) predicted no show–related surgery cancellation rate at Cincinnati Children’s Hospital Medical Center; (E) observed nil per os violation-related surgery cancellation rate at Cincinnati Children’s Hospital Medical Center; (F) predicted nil per os violation-related surgery cancellation rate at Cincinnati Children’s Hospital Medical Center; (G) observed patient or family refusal–related surgery cancellation rate at Cincinnati Children’s Hospital Medical Center; (H) predicted patient or family refusal–related surgery cancellation rate at Cincinnati Children’s Hospital Medical Center. CCHMC: Cincinnati Children’s Hospital Medical Center; NPO: nil per os; TCH: Texas Children’s Hospital; SCR: surgery cancellation rate.

The predicted DoSC rates at the census tract level from the best-performing models for CCHMC and TCH are compared with the actual data in the maps presented in Figure 7. Areas in proximity to hospital locations and with larger populations showed a higher correlation between the observed and predicted DoSC rates. Figure 7 shows the observed and predicted DoSC rates for specific cancellation causes, as detailed in the next section (enlarged maps in Figures S4-S11 in Multimedia Appendix 1). The interactive map versions of Figure 7 are presented in Multimedia Appendix 2 [50].

### Predicting Specific Cancellation Codes

Cancellations were coded by reason in the CCHMC data set. The four most frequent DoSC reasons account for more than 87% of the cases. These are patient illness (40%), nonattendance (*no show*; 20%), concern for aspiration risk because of noncompliance with preoperative *NPO* instructions (NPO violation; 18%), and patient or family refusing surgery after scheduling (10%) [6]. Cancellation because of patient illness was not analyzed using a spatial approach because there was no evidence of significant geospatial clustering (Figure 3). Spatial risk analyses and predicted cancellation rates generated by the best-performing models (Figure S12 in Multimedia Appendix 1) for the other individual cancellation codes are presented in Figure 7. Models using EHR data and ACS data aggregated at census tract level achieved better performance than individual-prediction-aggregation models (RMSEs of 1.124%, 0.868%, and 0.517% for *no show*–related, *NPO violation*–related, and *patient or family refusal*–related surgery cancellations, respectively). Of the 3 individual cancellation causes examined in this way, *no show* showed better geographically weighted Pearson correlation between observed and predicted DoSC rates than NPO violation or patient or family refusal (Figure 7). The key features for the specific cancellation reasons are shown in Figure 5. Prior cancellation behaviors, including the number of previous *no show* cancellations and clinic appointment *no shows* in the previous 6 months were predictive for day-of-surgery *no show* cancellation rates. Among community-level factors, the proportion of never married adults showed an association with *no show* cancellation rates. For both NPO violation–related and patient or family refusal–related cancellation rates, the proportion of African Americans per census tract, as well as ACS markers of poverty (including the proportions of households without a car, families in poverty or renting homes, median household income, and median house value) were salient. Linguistic isolation was predictive of the rates of cancellation because of patient or family refusal.

## Discussion

### Principal Findings

This study aims to understand the potential contributors to disparities in DoSC rates across neighborhoods. At 2 different tertiary children’s hospitals, we found marked geographic variation, particularly for cancellations coded as unrelated to patient illness. To understand this spatial variation, we developed models using case-based EHR data and ACS data aggregated at the census tract level. For the CCHMC data set, an L2-normalized GLM achieved the best performance in predicting the all-cause DoSC rate, but its improvement over the other regression models was marginal. The L2-normalized SAR model showed a comparable performance. The L2-normalized GLM performed better in urban areas around the CCHMC (Figure 7), possibly because of the larger number of surgical cases in these tracts. Interestingly, deep learning models did not offer improved predictive power, suggesting that geospatial impacts on DoSCs could be more regional and linear. A similar performance trend was observed for the TCH data set, suggesting the generalizability of our approach. Looking into the specific causes of cancellation at CCHMC, we found that patient illness (the most frequently recorded cause) did not show marked geographic variation. Of the 3 most frequent individual causes that showed spatial variation, *no show* was predicted better than NPO violation and patient or family refusal by the models.

Our geospatial analysis was helpful in identifying key factors, including potentially actionable predictors and underlying DoSCs at the census tract level. In the CCHMC data set (where the categorization of cancellation reasons was available from the EHR, unlike for TCH), the all-cause DoSC rate is composed of the top four most frequent patient-related cancellation causes (ie, patient illness, *no show*, NPO violation, and patient or family refusal). The key factors for all-cause cancellation reflect the average consensus for specific cancellation causes. The rate of prior *no show* cancellations by patients in a census tract best predicted the all-cause DoSC rate (Figure 5). The use of ACS data also provided granular and relevant information on community social and economic factors that were not available from the EHR, expanding the view of contextual factors likely influencing a family’s preparations for their child’s surgery [51]. We found that the proportion of African American inhabitants per census tract was predictive of geographic variation in the all-cause cancellation rate; that is, tracts with a higher proportion of African American inhabitants had higher DoSC rates. Important predictors for the 3 individual cancellation causes are discussed in detail below.

For the best model to predict *no show* cancellation rate, prior cancellation behaviors were of prime importance, including the number of previous *no show* cancellations and clinic *no show* in the previous 6 months (Figure 5). Both the GLM and SAR model suggested that every unit increase in the number of previous *no shows* was associated with a ~30% increase in the *no show* DoSC rate (Figures S13 and S14 in Multimedia Appendix 1). These patient- or family-level factors were complemented by a series of contextual variables that proved relevant. Indeed, for *no show* DoSCs, we found a similar link with the census tract proportion of adults who had never married, a potential surrogate for single parent–headed households. We speculate that such households experience more difficulty navigating day-to-day schedules. Working single parents may have trouble taking time off work. Those with multiple children may be challenged to ensure adequate coverage for their care. Obtaining appointments can be difficult; they may be especially difficult for those without a robust support structure [52].

Turning to NPO violation–related and patient or family refusal–related cancellations, we found commonality in key predictors. The proportion of African American inhabitants per census tract was predictive of both NPO violation–related and patient or family refusal–related DoSC rates. There are a variety of reasons that could underlie this finding—the reasons built atop analogous findings that highlight racial disparities across a range of health outcomes [53-56]. Racial segregation is strongly correlated with socioeconomic segregation in the United States. We found that, in addition to the proportion of African Americans within a tract, median household income, median house value, proportion of households without a car, and the proportion of families in poverty or renting homes were also influential [57,58]. Thus, it is possible that the link between race and DoSC was largely driven by structural racism and the concomitant challenges that accompany the disproportionate economic disadvantage experienced by racial minorities (eg, lack of trust in the health care system, inflexible work schedules, transportation barriers, and competing priorities) [59-61]. Moreover, the proportion of adults who were never married was salient to both. Linguistic isolation was associated with higher rates of cancellation because of patient or family refusal (Figure 5). We, therefore, hypothesize that CCHMC’s communications with urban, poor, minority, and non–English-speaking families leave room for improvement. Similar predictors (in this case for all-cause cancellation) were also identified using the TCH data set, including the proportion of African Americans per census tract and ACS markers of poverty (proportion of overcrowded households, families renting homes, low median household income, and low median house value; Figure 6).

Our analysis is novel for demonstrating that surgery cancellation is a source of inequity in surgical care and also for applying geospatial analysis to investigate barriers to care delivery. Furthermore, unlike the majority of studies in the literature on surgery cancellation, our study investigates patient- and family-related factors in the community using a geographic data set and offers insight into the underlying spatial risk factors and barriers experienced by families.

The findings of this study offer encouragement that geospatial analysis could appropriately be used to target interventions for patients living in census tracts with a higher rate of cancellations. In this way, support can be focused efficiently on families who are both in need and are most likely to benefit. Moreover, the specific predictors identified for individual cancellation codes may inform the design of interventions to address specific failure modes. For example, the association of cancellation risk with linguistic isolation argues for delivering preoperative communications using clear and simple language and for ready availability of interpreting services.

Arguably, our results uniquely reflect the characteristics of the Greater Cincinnati conurbation and its surrounding area, the pattern of referrals for surgery at CCHMC, and preoperative processes at this hospital. Thus, the findings may not be directly applicable to other locations. However, we hypothesize that similar social factors may determine cancellation patterns in other hospitals offering surgery for children, as evidenced by the similar performance and predictors observed in the TCH data set. In particular, the methodology used is likely to be transferable to other locales and institutions and to disparate aspects of health care delivery. With the easy availability of high-quality commercial or open-sourced geocoding software, our approach will be relatively easy to translate.

### Limitations

We acknowledge that our study is limited in several ways. First, as an observational study, *exposures* to socioeconomic disadvantage or to racial minority status are not the only potential explanations for observed differences in cancellation rates. Second, the study relied on extracting patients’ home addresses from the EHRs, which may be inaccurate (eg, outdated) or incomplete. In addition, cancellation of children’s surgery likely depends on individual circumstances and perhaps seasonal factors. Finally, the relatively low number of census tracts (sample size) in the data sets might limit the application of complex models, such as deep learning.

### Conclusions

This study aimed to conduct a geospatial analysis of patient-specific variables from EHRs and linked socioeconomic factors to understand the underlying contributors to disparities in DoSC rates across neighborhoods. Our findings demonstrate the importance of home location, socioeconomic disadvantage, and racial minority status on the last-minute cancellation of children’s surgery. The success of future efforts to reduce cancellation may benefit from taking social, economic, and cultural issues into account. Although the original aim of this study was to drive improvement efforts, our results add further evidence of the importance of social determinants in children’s health, including increased incidence and frequency of illness, barriers to accessing health care, and readmissions [62,63].

## Appendix

Multimedia Appendix 1 Supplementary figures of enlarged maps, feature importance, and model performance.

Multimedia Appendix 2 Interactive maps.

## Abbreviations

ACS: American Community Survey
CCHMC: Cincinnati Children’s Hospital Medical Center
CNN: convolutional neural network
DALEX: Model Agnostic Language for Exploration and Explanation
DoSC: day-of-surgery cancellation
EHR: electronic health record
GCN: graph convolutional network
GLM: generalized linear regression model
NPO: nil per os
RMSE: root mean square error
SAR: spatial autoregressive
SEM: spatial error model
SVM-P: support vector machine with polynomial kernels
TCH: Texas Children’s Hospital
VIF: variance inflation factor
