# Supplementary material for: Understanding Pediatric Surgery Cancellation: Geospatial Analysis
Source: J Med Internet Res. 2021 Sep 10;23(9):e26231. doi: 10.2196/26231 (PMC8463951; doi:10.2196/26231)
Supplement: Multimedia Appendix 1 [file jmir_v23i9e26231_app1.docx]

**Geospatial Analysis to Understand Pediatric Surgery Cancellation**

Lei Liu, BS^1, 2^, Yizhao Ni, PhD^1, 3^, Andrew F. Beck, MD, MPH^3, 4^, Cole Brokamp, PhD^3, 5^, Ryan C. Ramphul, PhD^6^, Linda D. Highfield, PhD^7^, Megha Karkera Kanjia, MD^8, 9^, J. “Nick” Pratap, MB BChir, MRCPCH, FRCA^3, 10^

Affiliations:

1. Division of Biomedical Informatics, Cincinnati Children’s Hospital Medical Center, Cincinnati, Ohio, USA

2. Department of Biomedical Informatics, University of Cincinnati, College of Medicine, Cincinnati, Ohio, USA.

3. Department of Pediatrics, University of Cincinnati, College of Medicine, Cincinnati, Ohio, USA.

4. Divisions of General and Community Pediatrics and Hospital Medicine, Cincinnati Children’s Hospital Medical Center, Cincinnati, Ohio, USA.

5. Divisions of Biostatistics and Epidemiology, Cincinnati Children’s Hospital Medical Center, Cincinnati, Ohio, USA.

6. Department of Government Relations and Community Benefits, Texas Children’s Hospital, Houston, Texas, USA.

7. Department of Management, Policy and Community Health Practice and Epidemiology, University of Texas Health Science Center School of Public Health, Houston, Texas, USA.

8. Department of Pediatric Anesthesiology and Pain Management, Texas Children’s Hospital, Houston, Texas, USA.

9. Department of Anesthesiology, Baylor College of Medicine, Houston, Texas, USA.

10. Department of Anesthesia, Cincinnati Children's Hospital Medical Center, Cincinnati, Ohio, USA.

*Corresponding Author: J. “Nick” Pratap, MB BChir, MRCPCH, FRCA

Department of Anesthesia, MLC 2001

Cincinnati Children's Hospital Medical Center

3333 Burnet Avenue

Cincinnati, OH 45229-3039

Phone: 513-636-4408

Email: jayant.pratap@cchmc.org


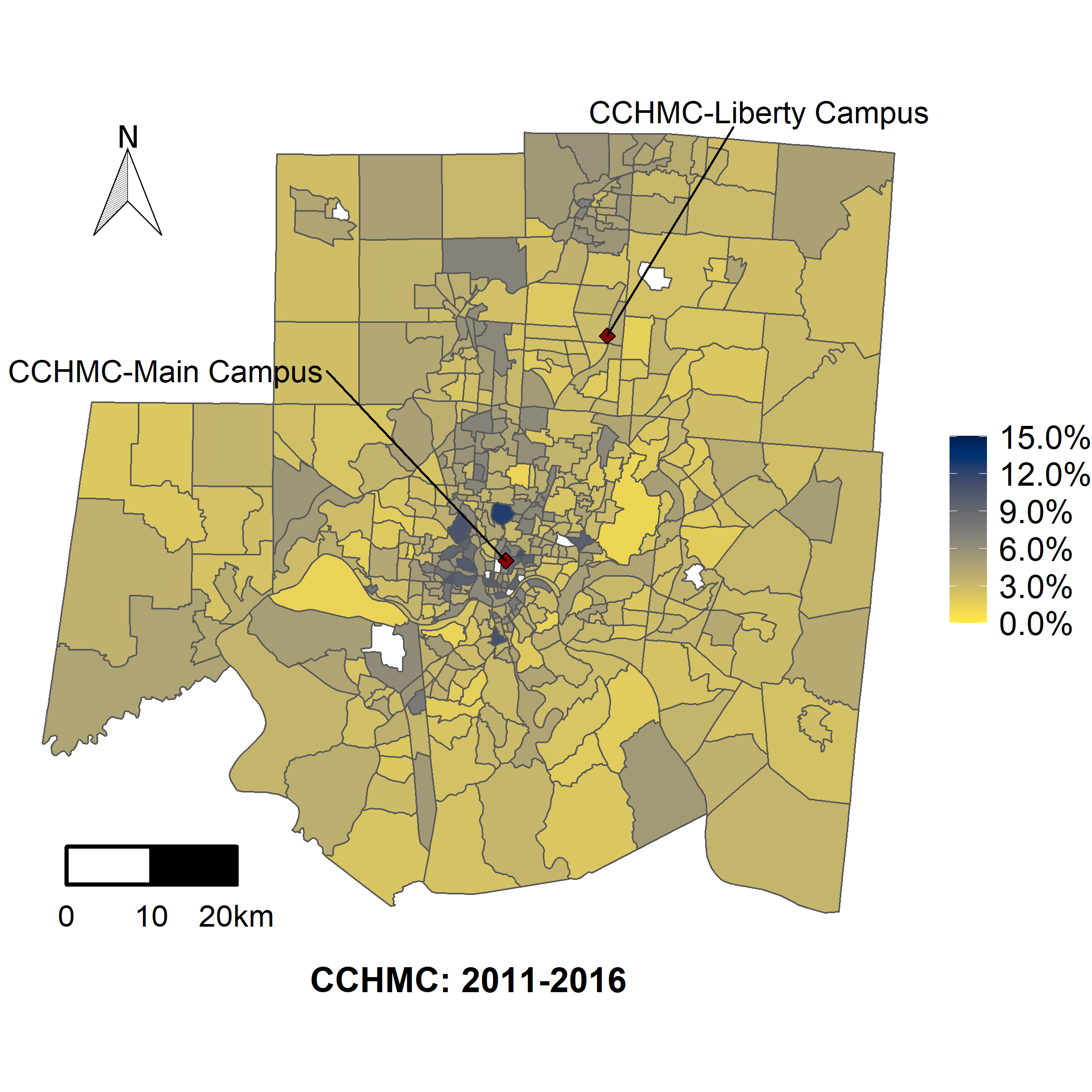


**Figure S1.** Enlarged version of Figure 2A which presents the geospatial distributions of all-cause surgery cancellation rate in the primary service area of CCHMC.


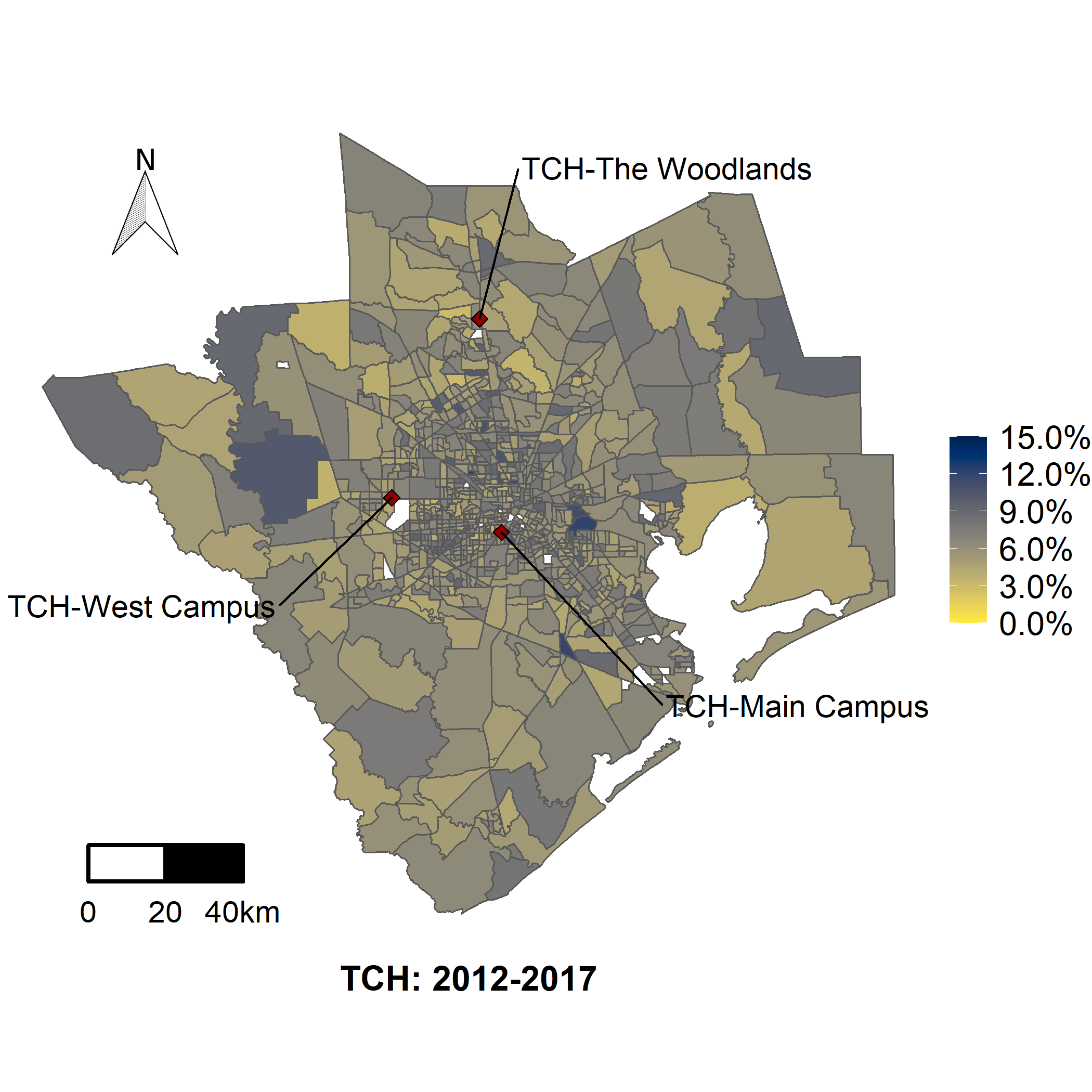


**Figure S2.** Enlarged version of Figure 2B which presents the geospatial distributions of surgery cancellation rate in the primary service area of TCH.


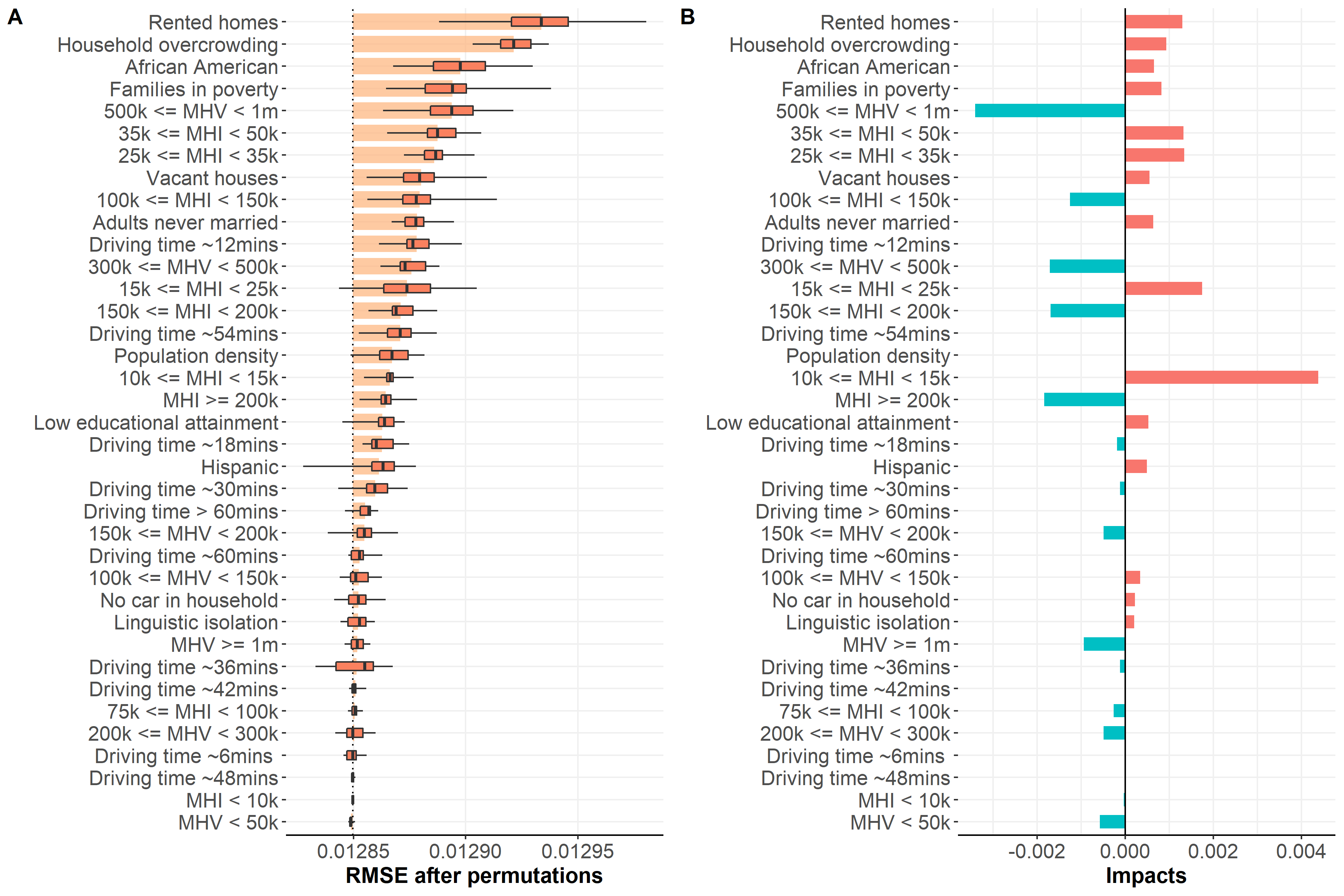


**Figure S3** A) Feature importance and B) impacts generated from the best performed L2-normalized SAR model for TCH. Abbreviations: MHI = median household income, MHV = median house value.


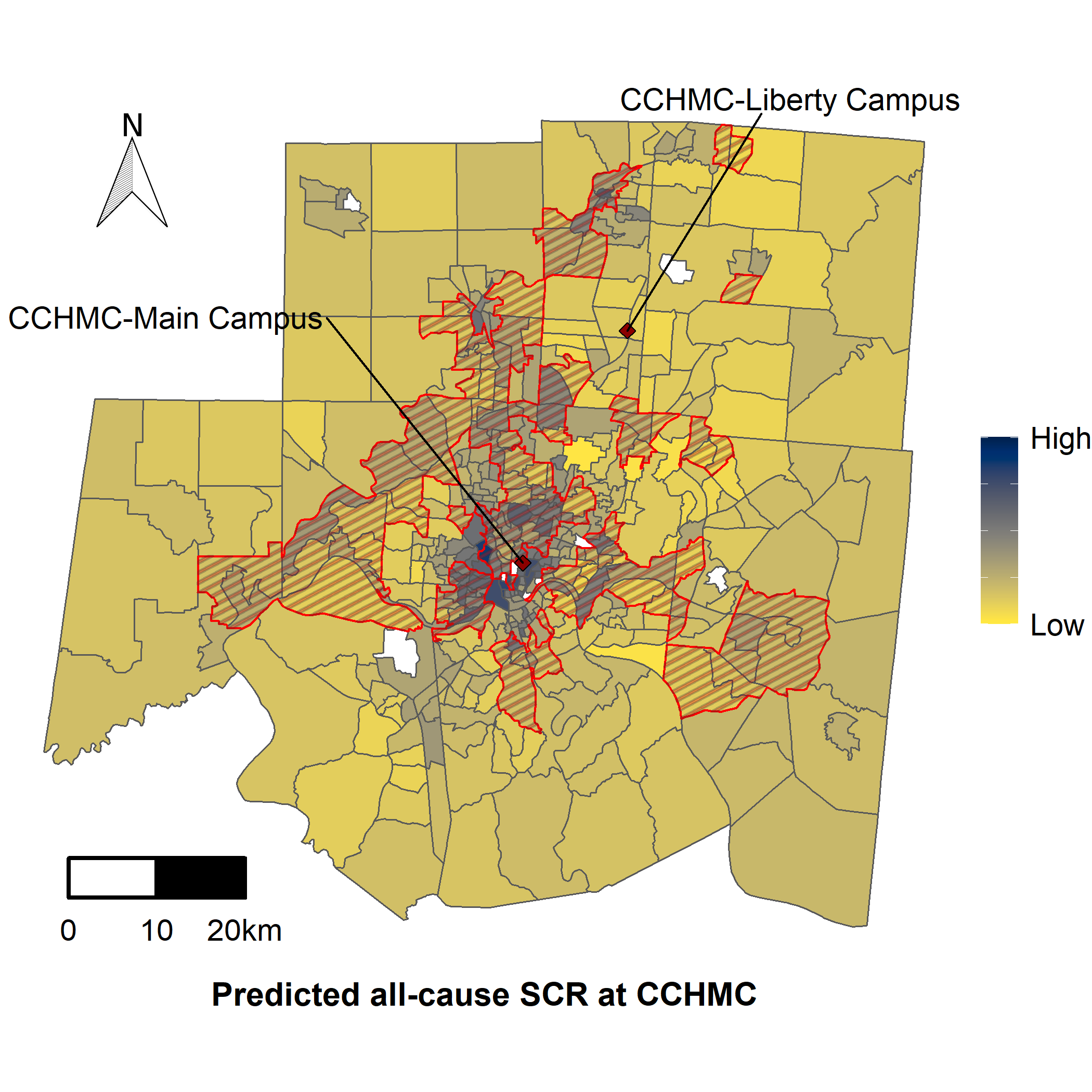


**Figure S4.** Enlarged version of Figure 7A which presents the predicted all-cause surgery cancellation rates at CCHMC. Census tracts with significant local correlation between the observed and predicted values are marked with red borders and cross-hatching. Abbreviations: SCR = surgery cancellation rate


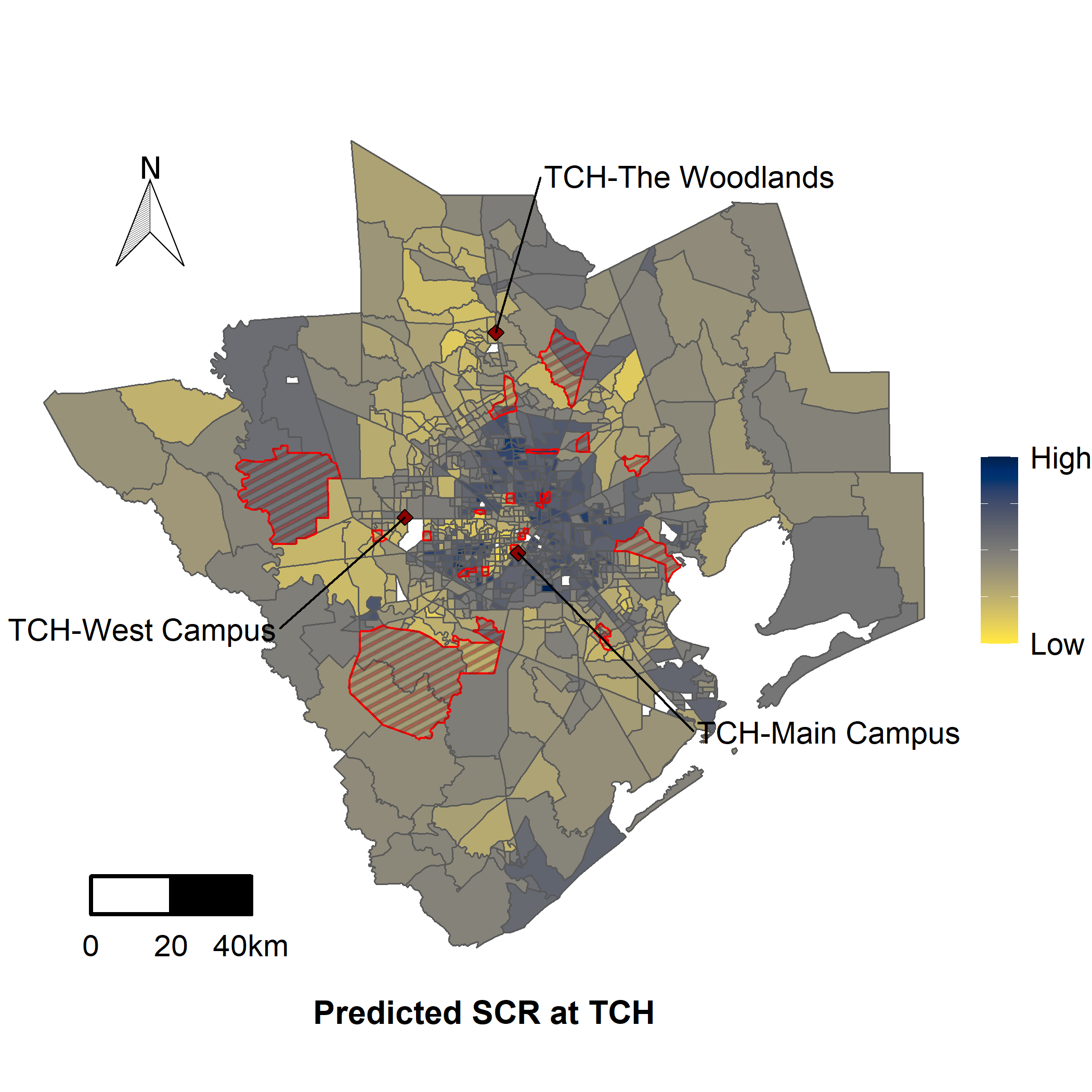


**Figure S5.** Enlarged version of Figure 7B which presents the predicted surgery cancellation rates at TCH. Census tracts with significant local correlation between the observed and predicted values are marked with red borders and cross-hatching. Abbreviations: SCR = surgery cancellation rate


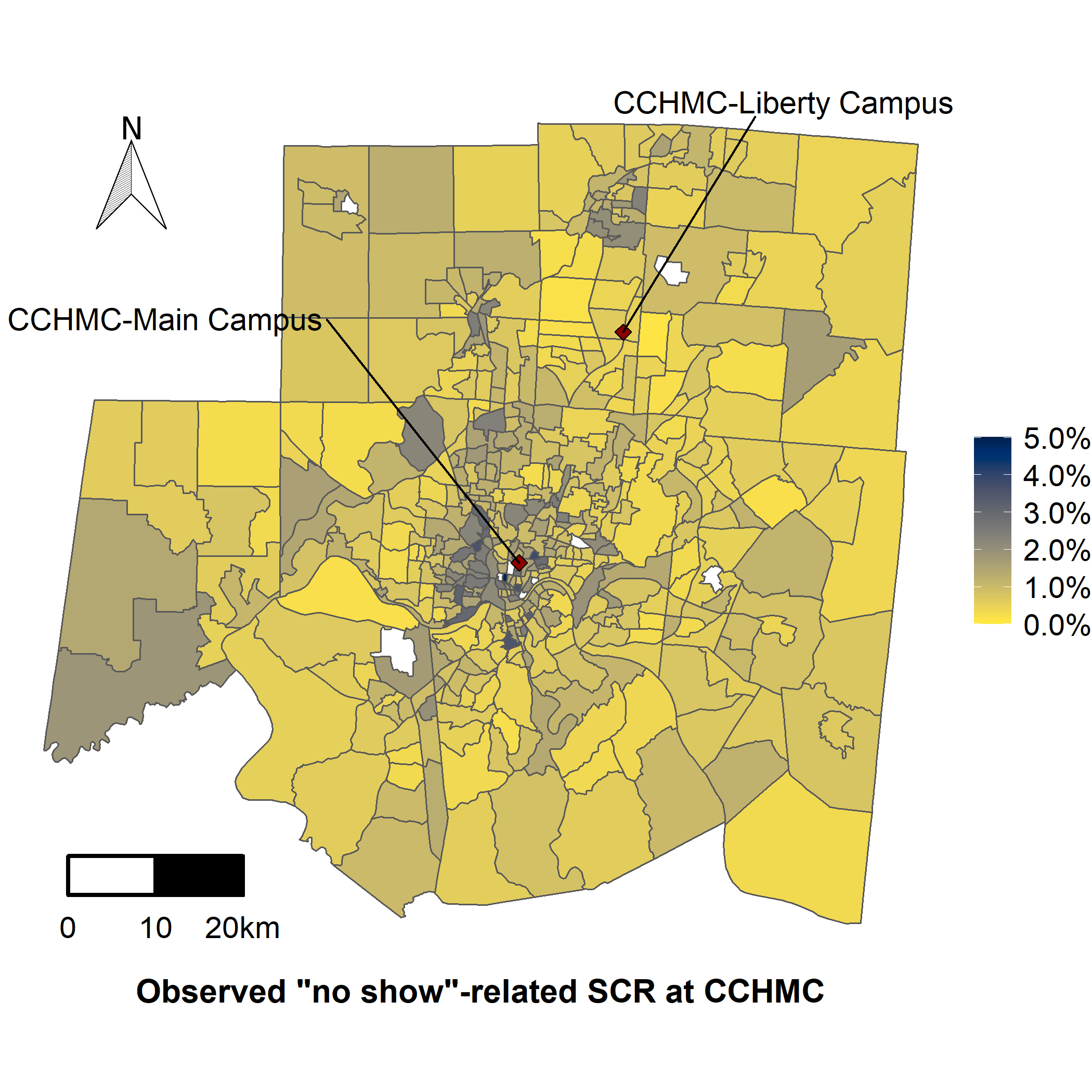


**Figure S6.** Enlarged version of Figure 7C which presents the observed “no-show”-related surgery cancellation rates at CCHMC. Abbreviations: SCR = surgery cancellation rate


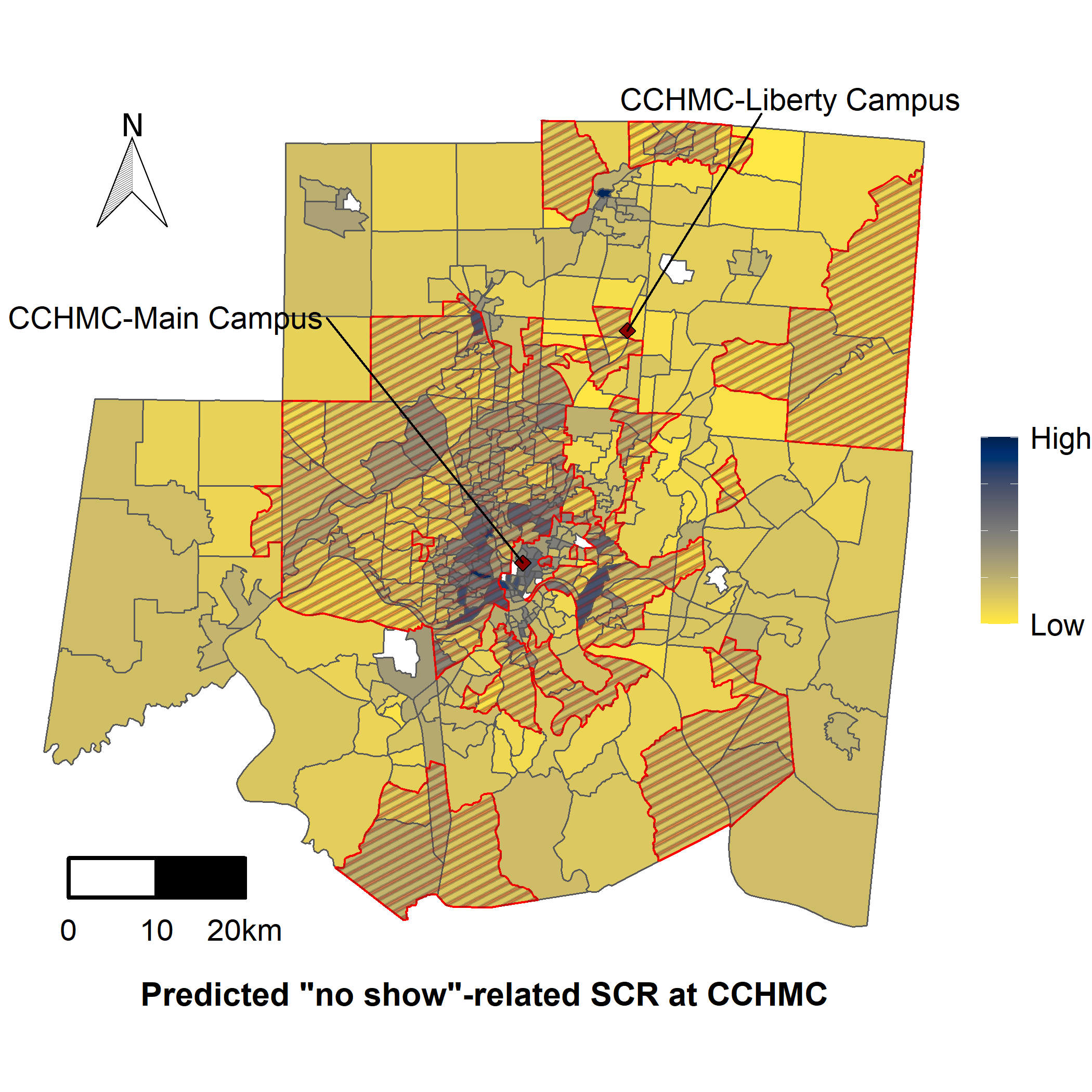


**Figure S7.** Enlarged version of Figure 7D which presents the predicted “no show”-related surgery cancellation rates at CCHMC. Census tracts with significant local correlation between the observed and predicted values are marked with red borders and cross-hatching. Abbreviations: SCR = surgery cancellation rate


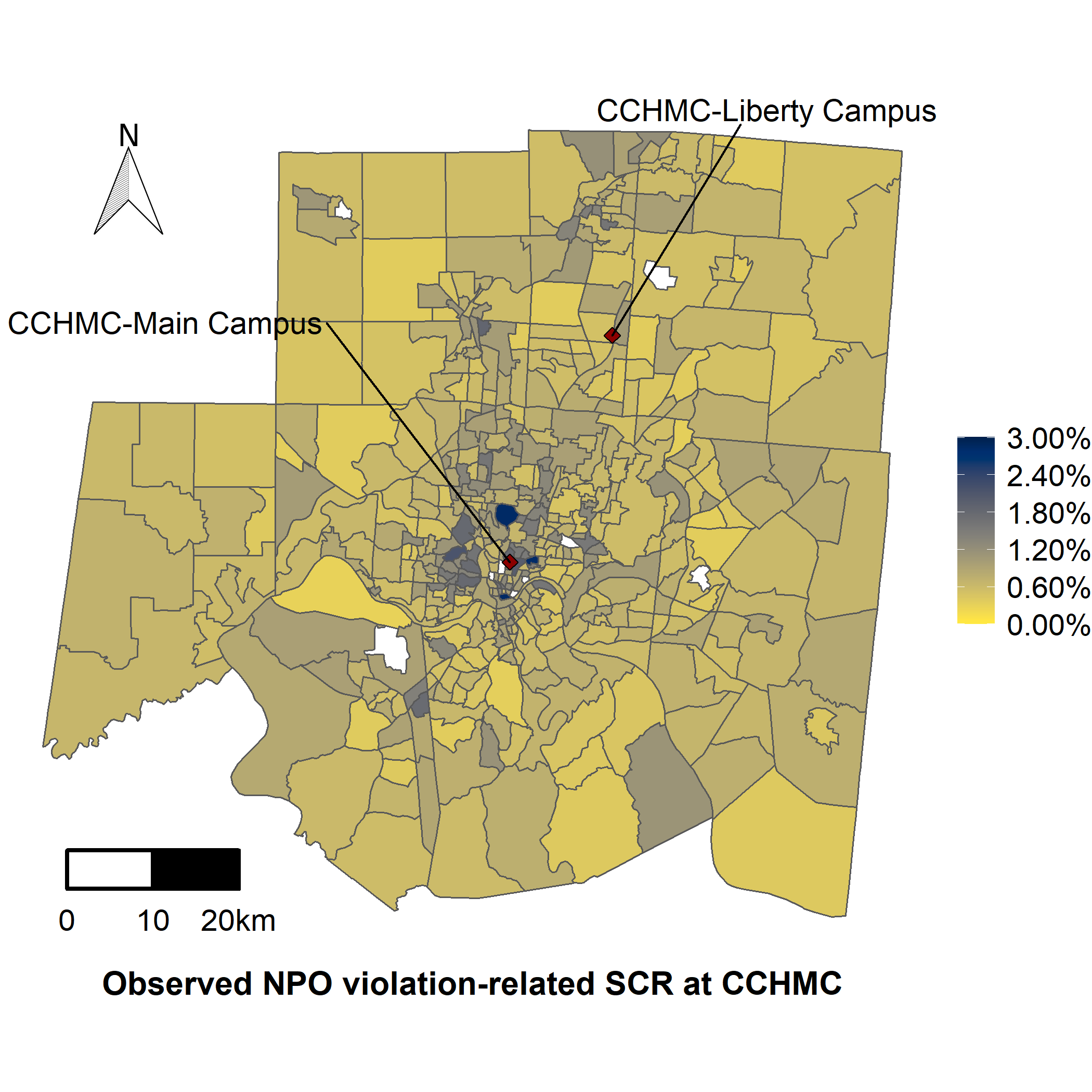


**Figure S8.** Enlarged version of Figure 7E which presents the observed NPO violation-related surgery cancellation rates at CCHMC. Abbreviations: SCR = surgery cancellation rate, NPO = *nil per os*


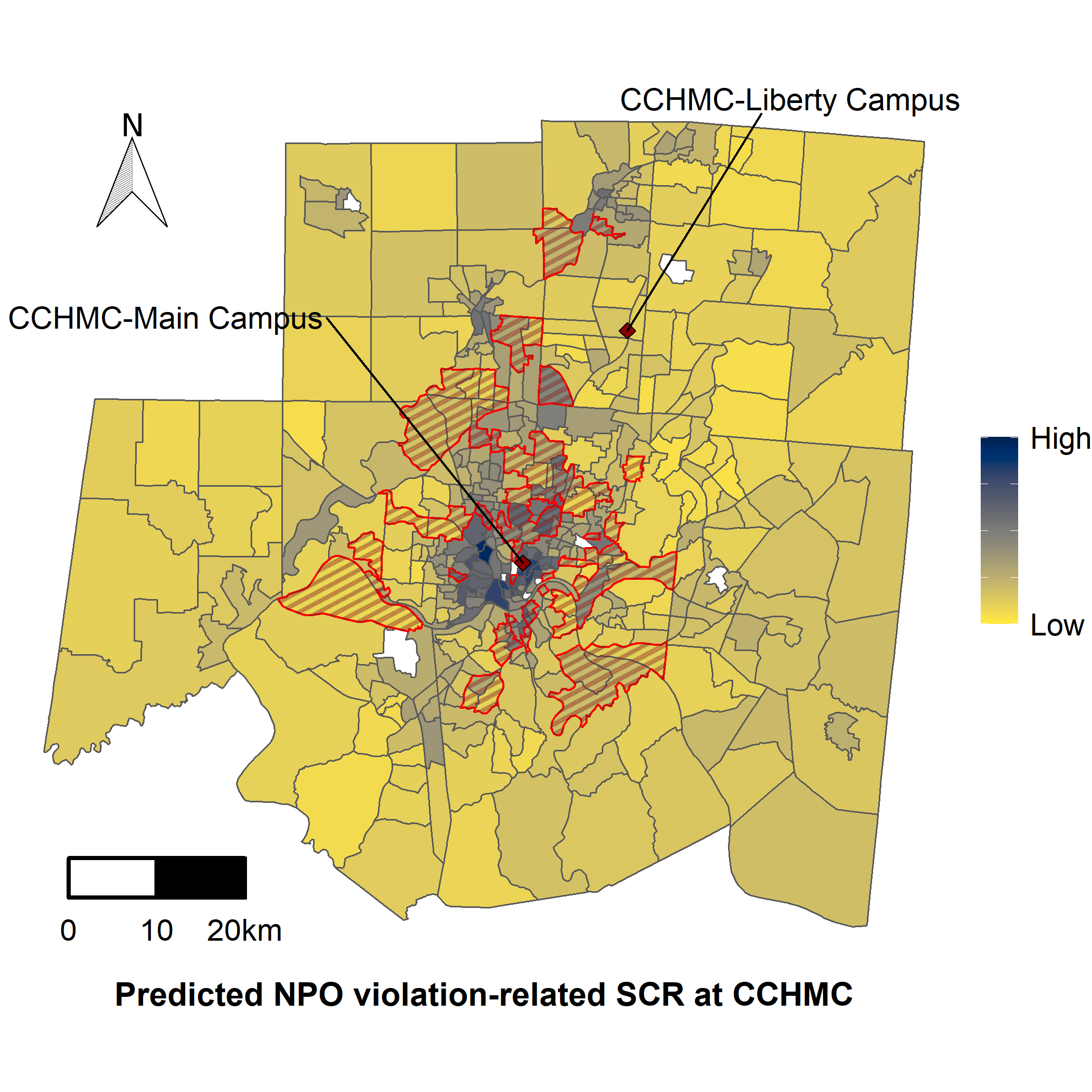


**Figure S9.** Enlarged version of Figure 7F which presents the predicted NPO violation-related surgery cancellation rates at CCHMC. Census tracts with significant local correlation between the observed and predicted values are marked with red borders and cross-hatching. Abbreviations: SCR = surgery cancellation rate, NPO = *nil per os*


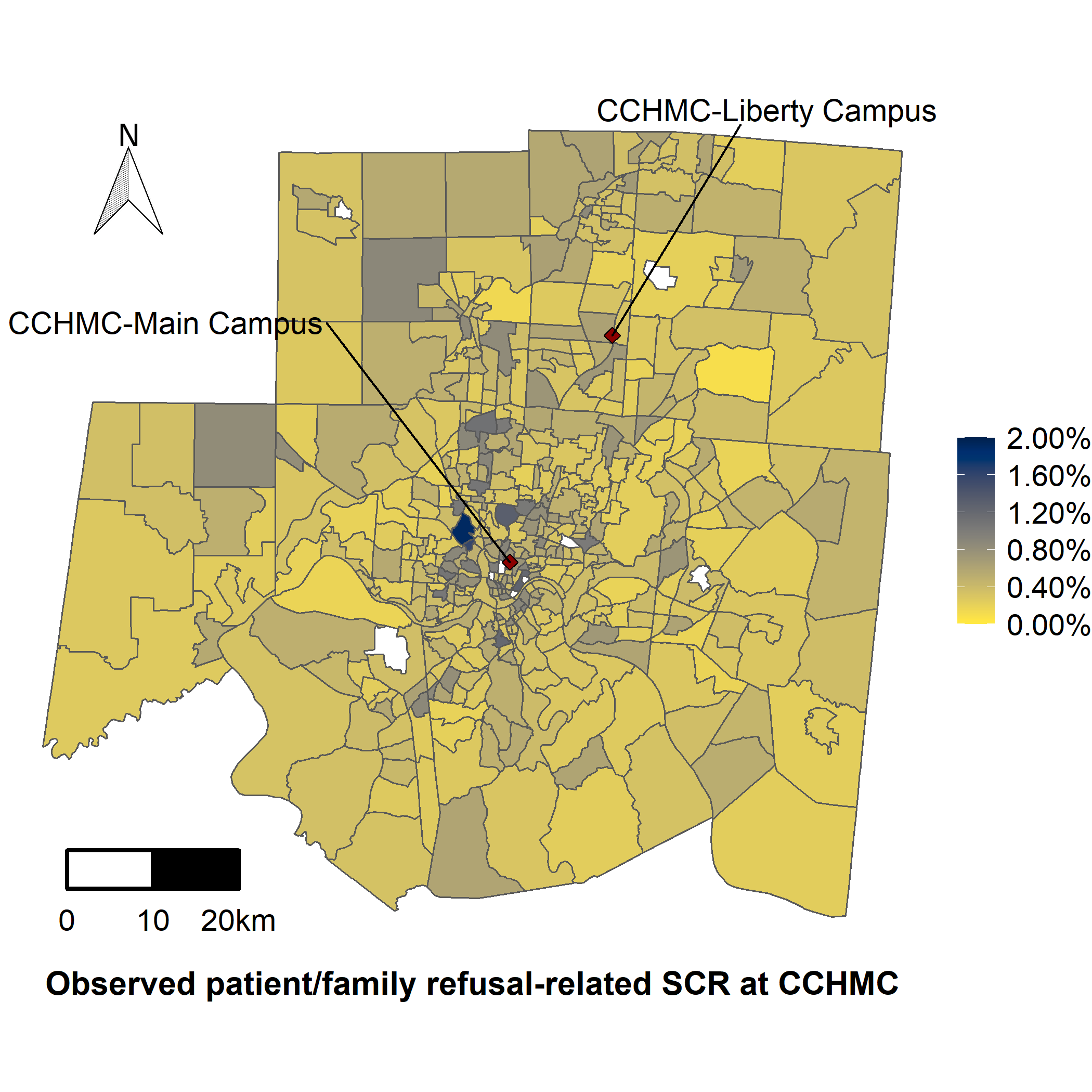


**Figure S10.** Enlarged version of Figure 7G which presents the observed patient/family refusal-related surgery cancellation rates at CCHMC. Abbreviations: SCR = surgery cancellation rate


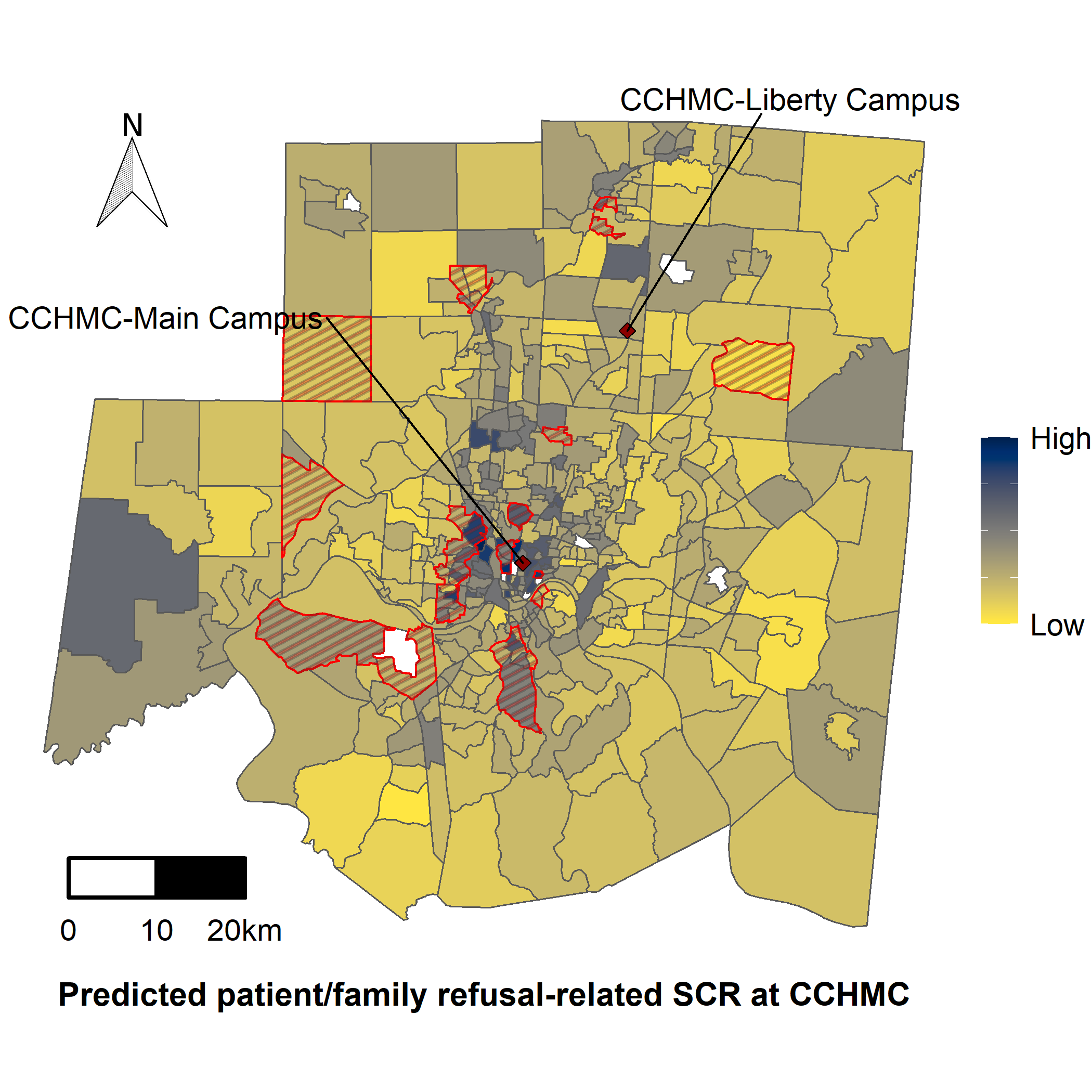


**Figure S11.** Enlarged version of Figure 7H which presents the predicted patient/family refusal-related surgery cancellation rates at CCHMC. Census tracts with significant local correlation between the observed and predicted values are marked with red borders and cross-hatching. Abbreviations: SCR = surgery cancellation rate


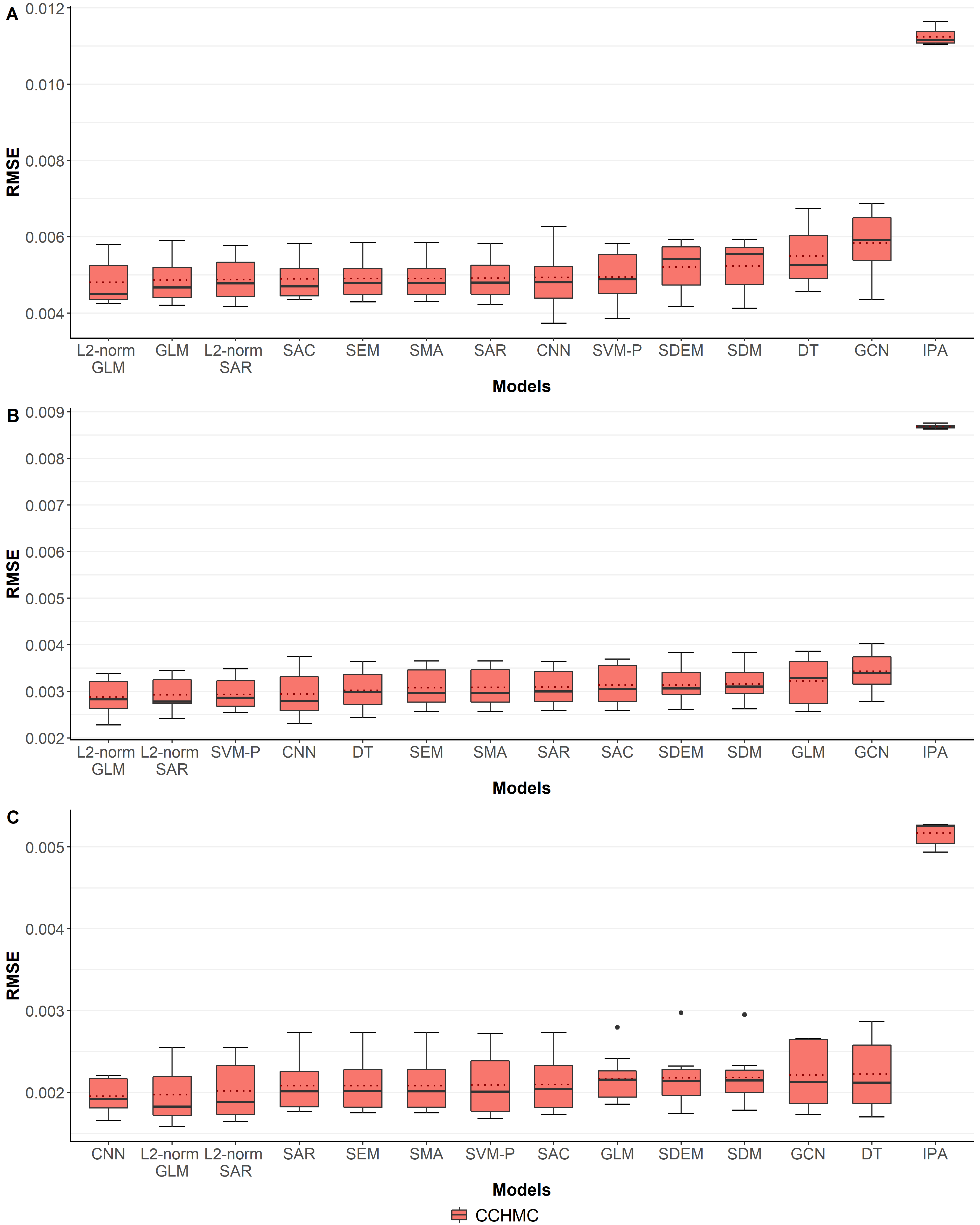


**Figure S12.** Model performance for predicting specific cancellation reasons at census tract level for CCHMC, including A) “no show”-, B) NPO violation- and C) patient/family refusal-related cancellation.


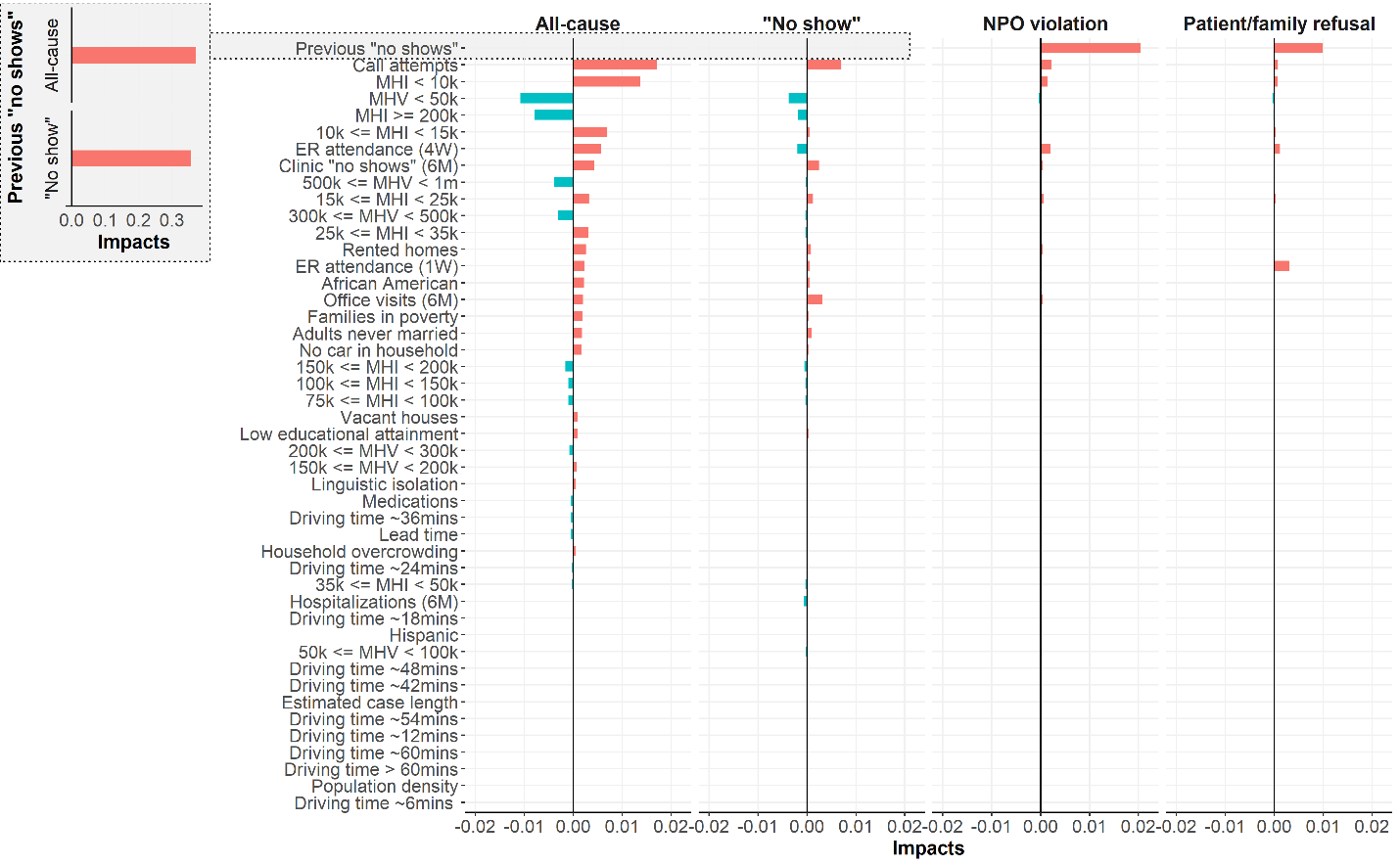


**Figure S13.** Impacts generated from the best performed L2-normalized GLM model for CCHMC. Abbreviations: NPO violation = failure to comply with eating/drinking instructions, ER = emergency room, MHI = median household income, MHV = median house value.


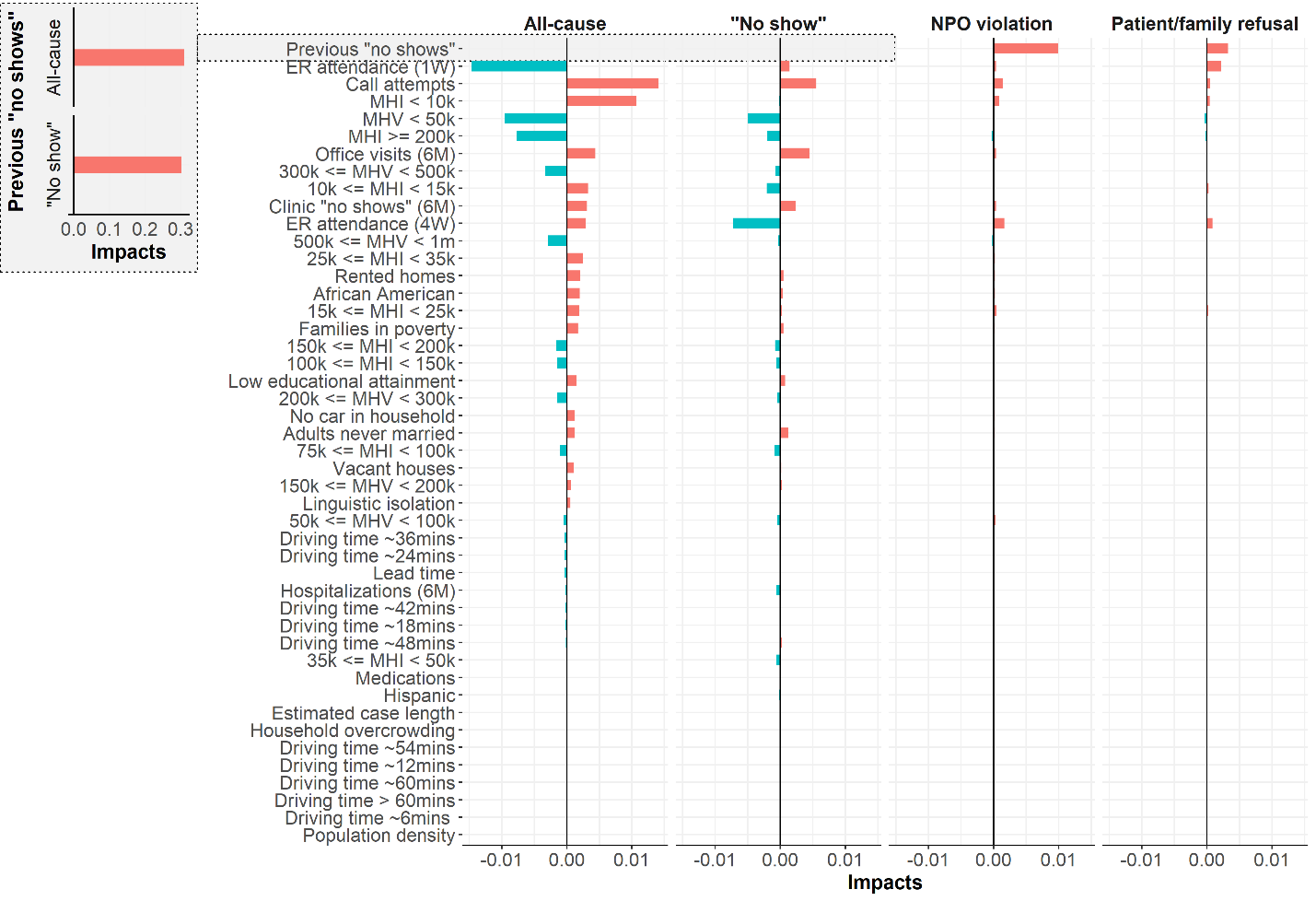


**Figure S14.** Impacts generated from the best performed L2-normalized SAR model for CCHMC. Abbreviations: NPO violation = failure to comply with eating/drinking instructions, ER = emergency room, MHI = median household income, MHV = median house value.
